# Long-term conservation tillage with reduced nitrogen fertilization intensity can improve winter wheat health via positive plant–microorganism feedback in the rhizosphere

**DOI:** 10.1093/femsec/fiae003

**Published:** 2024-01-15

**Authors:** Jan Helge Behr, Theresa Kuhl-Nagel, Loreen Sommermann, Narges Moradtalab, Soumitra Paul Chowdhury, Michael Schloter, Saskia Windisch, Ingo Schellenberg, Lorrie Maccario, Søren J Sørensen, Michael Rothballer, Joerg Geistlinger, Kornelia Smalla, Uwe Ludewig, Günter Neumann, Rita Grosch, Doreen Babin

**Affiliations:** Leibniz Institute of Vegetable and Ornamental Crops (IGZ), Plant-Microbe Systems, Theodor-Echtermeyer-Weg 1, 14979 Großbeeren, Germany; Leibniz Institute of Vegetable and Ornamental Crops (IGZ), Plant-Microbe Systems, Theodor-Echtermeyer-Weg 1, 14979 Großbeeren, Germany; Anhalt University of Applied Sciences, Department of Agriculture , Ecotrophology and Landscape Development, Strenzfelder Allee 28, 06406 Bernburg, Germany; University of Hohenheim, Institute of Crop Science (340 h), Fruwirthstraße 20, 70599 Stuttgart, Germany; Institute of Network Biology , Helmholtz Zentrum München, German Research Center for Environmental Health (GmbH), Ingolstaedter Landstraße 1, 85764 Neuherberg, Germany; Research Unit for Comparative Microbiome Analysis (COMI), Helmholtz Zentrum München, German Research Center for Environmental Health (GmbH), Ingolstaedter Landstraße 1, 85764 Neuherberg, Germany; University of Hohenheim, Institute of Crop Science (340 h), Fruwirthstraße 20, 70599 Stuttgart, Germany; Anhalt University of Applied Sciences, Department of Agriculture , Ecotrophology and Landscape Development, Strenzfelder Allee 28, 06406 Bernburg, Germany; University of Copenhagen, Department of Biology, Section of Microbiology, Universitetsparken 15, 2100 Copenhagen, Denmark; University of Copenhagen, Department of Biology, Section of Microbiology, Universitetsparken 15, 2100 Copenhagen, Denmark; Institute of Network Biology , Helmholtz Zentrum München, German Research Center for Environmental Health (GmbH), Ingolstaedter Landstraße 1, 85764 Neuherberg, Germany; Anhalt University of Applied Sciences, Department of Agriculture , Ecotrophology and Landscape Development, Strenzfelder Allee 28, 06406 Bernburg, Germany; Julius Kühn Institute (JKI) – Federal Research Centre for Cultivated Plants, Institute for Epidemiology and Pathogen Diagnostics, Messeweg 11-12, 38104 Braunschweig, Germany; University of Hohenheim, Institute of Crop Science (340 h), Fruwirthstraße 20, 70599 Stuttgart, Germany; University of Hohenheim, Institute of Crop Science (340 h), Fruwirthstraße 20, 70599 Stuttgart, Germany; Leibniz Institute of Vegetable and Ornamental Crops (IGZ), Plant-Microbe Systems, Theodor-Echtermeyer-Weg 1, 14979 Großbeeren, Germany; Julius Kühn Institute (JKI) – Federal Research Centre for Cultivated Plants, Institute for Epidemiology and Pathogen Diagnostics, Messeweg 11-12, 38104 Braunschweig, Germany

**Keywords:** 16S rRNA gene, ITS Illumina amplicon sequencing, mineral fertilization, root exudates, shotgun metagenome sequencing, sustainable agriculture

## Abstract

Microbiome-based solutions are regarded key for sustainable agroecosystems. However, it is unclear how agricultural practices affect the rhizosphere microbiome, plant–microorganism interactions and crop performance under field conditions. Therefore, we installed root observation windows in a winter wheat field cultivated either under long-term mouldboard plough (MP) or cultivator tillage (CT). Each tillage practice was also compared at two nitrogen (N) fertilization intensities, intensive (recommended N-supply with pesticides/growth regulators) or extensive (reduced N-supply, no fungicides/growth regulators). Shoot biomass, root exudates and rhizosphere metabolites, physiological stress indicators, and gene expression were analyzed together with the rhizosphere microbiome (bacterial/archaeal 16S rRNA gene, fungal ITS amplicon, and shotgun metagenome sequencing) shortly before flowering. Compared to MP, the rhizosphere of CT winter wheat contained more primary and secondary metabolites, especially benzoxazinoid derivatives. Potential copiotrophic and plant-beneficial taxa (e.g. *Bacillus, Devosia*, and *Trichoderma*) as well as functional genes (e.g. siderophore production, trehalose synthase, and ACC deaminase) were enriched in the CT rhizosphere, suggesting that tillage affected belowground plant–microorganism interactions. In addition, physiological stress markers were suppressed in CT winter wheat compared to MP. In summary, tillage practice was a major driver of crop performance, root deposits, and rhizosphere microbiome interactions, while the N-fertilization intensity was also relevant, but less important.

## Teaser

Cultivator tillage combined with reduced nitrogen fertilization intensity resulted in a positive plant–microorganism feedback in the rhizosphere contributing to an improved plant performance compared to conventional management.

## Introduction

The increasing demands for plant-based products together with the reduced availability of sites for agriculture has led to more intensive farming practice over the last decades (Hoang et al. 2023). This intensification, often achieved by high use of agrochemicals, has negative consequences for soil quality and overall ecosystem functions (Kopittke et al. 2019, Timmis and Ramos 2021). To counteract the increasing degradation of soils, less intensive, so-called conservation farming practices including diverse crop rotations, reduced tillage combined with lower pesticide and fertilizer use have been promoted (Hobbs et al. 2008).

Soil microorganisms play an essential role for soil structure and fertility (Fierer 2017, Banerjee and van der Heijden 2023). Furthermore, soil microorganisms represent the reservoir from which the plant assembles its rhizosphere (RH) microbiome (Philippot et al. 2013). The RH, i.e. the carbon-enriched thin layer of soil surrounding roots and influenced by root activity (Berg and Smalla 2009), is a hot spot for microbial activity and is considered one of the most complex and diverse ecosystems (Hinsinger et al. 2009, Raaijmakers et al. 2009). The RH microbiome plays a pivotal role in plant development and health (Berendsen et al. 2012, Mendes et al. 2013). For instance, an improved plant tolerance to abiotic and biotic stressors can be the result of beneficial plant–microorganism interactions in the RH (Panke-Buisse et al. 2015, Mommer et al. 2016). Therefore, engineering the soil and RH microbiome is crucial for sustainable agroecosystems (Toju et al. 2018, Trivedi et al. 2021).

Plants have evolved various mechanisms to modulate their RH microbiome (Berendsen et al. 2012, Lareen et al. 2016), including the release of root exudates and other organic rhizodeposits, which can account for up to 11% of photosynthetically fixed carbon (Jones et al. 2009). Low molecular weight (LMW) root exudates, released either by diffusion or via controlled secretion, comprise the full range of primary metabolites as well as a wide range of secondary plant compounds. Generally, LMW sugars in root exudates represent an easily available carbon source for microorganisms. Exuded carboxylates such as malate and citrate are chemo-attractants and preferential carbon sources for N_2_-fixing RH bacteria. Moreover, they can act as metal chelators and can thereby contribute to the mobilization of micronutrients and sparingly soluble phosphorus (P) in soils. Additionally, carboxylates can neutralize toxic aluminum as well as adaptively modify root architecture (Canarini et al. 2019, Neumann and Ludewig 2023). Amino acids are important nitrogen (N) sources for microorganisms. Furthermore, they can function as precursors for microbial production of phytohormones, signals in adaptations for N acquisition and as metal chelators (Canarini et al. 2019, Neumann and Ludewig 2023). Exuded secondary metabolites comprise also diverse bioactive compounds (Philippot et al. 2013), such as benzoxazinoids (BXs; Kudjordjie et al. 2019, Cadot et al. 2021), coumarins (Stassen et al. 2020), flavones (Yu et al. 2021), triterpenes (Huang et al. 2019), and camalexin (Koprivova et al. 2019), which were found to contribute to root–microbiome interactions and various cross-kingdom relationships.

Previous studies demonstrated that the legacy of agricultural practices affect the RH microbiome (Sommermann et al. 2018, Babin et al. 2019, Cerecetto et al. 2021), and thereby the performance and health of plants (Chowdhury et al. 2019, Babin et al. 2021, Bourceret et al. 2022, Flemer et al. 2022). Using lettuce as a model in minirhizotrons with root observation windows, Neumann et al. (2014) and Windisch et al. (2021) showed that the soil type and the fertilization strategy affected the root exudation profiles and accumulation of RH metabolites likely as a result of plant interactions with the soil-specific microbiomes. However, realistic insights into the dynamics of root exudate releases and turnover under field conditions and related interactions with pathogenic and beneficial soil microorganisms remain largely unexplored (Kawasaki et al. 2018, Oburger et al. 2022). Consequently, factors influencing plant–microorganism interactions and processes in agroecosystems are not yet fully understood (Canarini et al. 2019).

In order to exemplarily address these knowledge gaps and provide a holistic understanding of soil–plant–microorganism interactions in agricultural settings, we installed root windows, similar to large rhizotrons, in winter wheat plots of a long-term field experiment managed under contrasting tillage types [cultivator (CT) vs. mouldboard plough (MP)] and intensities of N-fertilization and pesticide/growth regulator use [intensive (Int) vs. extensive (Ext)]. An interdisciplinary approach enabled us to assess root growth characteristics and RH metabolites *in situ* along with the soil and RH microbiome. Aboveground, shoot biomass, plant nutritional status, and physiological stress indicators were monitored to assess plant health.

## Material and methods

### Study site and experimental design

The long-term field trial in Bernburg (Germany; 51.82°N, 11.70°E), which was established at Anhalt University of Applied Sciences in 1992 on a loess chernozem over limestone (Deubel et al. 2011), served as the study site. The field trial consists of five 1.2 ha-sized plots, which are administered by a yearly crop rotation comprising grain maize (*Zea mays*), winter wheat 1 (*Triticum aestivum*), winter barley (*Hordeum vulgare*), winter rapeseed (*Brassica napus* ssp. *napus*), and winter wheat 2. Each plot is managed with conventional MP (20–30 cm ploughing depth, soil inversion) or conservation cultivator tillage (CT, 12–15 cm depth, flat soil loosening) under either intensive N-fertilization and pesticide/growth regulator application according to usual farming practice (Int) or reduced, extensive N-fertilization without addition of fungicides and growth regulators (Ext). Thus, on each plot four agricultural practices (MP-Int, MP-Ext, CT-Int, and CT-Ext) each in four replicates were compared. In this study, we focused on winter wheat 2 (cultivar Lemmy) in the season 2018/2019, which received until sampling 130 kg N ha^−1^ or 60 kg N ha^−1^ in Int or Ext, respectively (Text 1, Supporting Information).

Root windows were installed in spring 2019 at EC 29 in the marginal strips of each treatment (*n* = 4; Figure S1, Supporting Information; Neumann et al. 2009). In brief, 50-cm-deep soil profiles were cut with steel plates next to the young wheat plants and covered with plexiglass plates, which were fixed with square timbers. The windows were thermally isolated with two layers of Styrodur panels and covered with field soil and plastic sheets.

### Sampling

Approximately 6 weeks after root window installation and shortly before flowering (EC 59), sufficient root development was observed (Figure S2A, Supporting Information) and root windows were removed for microsampling of RH soil solutions (Windisch et al. 2021). Sampling at vegetative growth stage ensured high root exudation activity (Marschner 1995). Briefly, moist sorption filters (MN818, 5 Ø mm, Macherey & Nagel, Düren, Germany; Figure S2B, Supporting Information) were placed on apical root zones (two filters: 1–2 cm behind the root tip) and subapical root segments (two filters: 8–10 cm behind the root tip). For each root window, sampling was performed along five different roots growing at the observation plane. Furthermore, sorption filters were placed on soil without visible root contact [root-affected (RA) soil] in order to determine background noise. The sorption filters of each replicate and root zone were pooled after four hours of collection, immediately transferred to 2 ml methanol 80% (v/v) for stabilization and kept at −80°C until HPLC/HPLC-MS analyses for metabolite profiling (Windisch et al. 2021; see below).

After sorption filter collection, flag leaves of three different plants were sampled and pooled for plant gene expression, phytohormone and stress-related metabolite analysis (see below). The leaves were cut into small pieces (5 cm) and transferred in 15 ml tubes containing RNAlater solution (Ambion, Life technologies, Carlsbad, USA). The leaf tissue was stored overnight at 4°C to allow thorough RNAlater penetration and then transferred to −20°C for long-term storage.

For nutrient analysis, flag leaves of three plants were sampled and oven-dried at 60°C for 3 days and subsequently stored in a desiccator for another 2 days (see below).

For standardized root sampling, six winter wheat plants were excavated from each root window and divided into shoot and root. RA soil, defined as soil loosely adhering to roots, was obtained by shaking of roots and subsequently sieved to 2 mm and stored at −20°C until total microbial community (TC)-DNA extraction. For one replicate of treatment CT-Int, no RA soil was mistakenly sampled resulting in this case in only three instead of four replicates. For RH microbiome analysis we used complete root systems of winter wheat, from which, after brief washing with sterile tap water, microbial cells and strongly adhering soil particles were recovered from 5 g roots by three times 1 min Stomacher treatment and centrifugation according to Schreiter et al. (2014). The resulting RH pellets were kept at −20°C until total microbial community (TC)-DNA extraction (see below). Roots after Stomacher treatment were stored at 4°C until determination of root parameters (see below).

### Determination of above- and belowground plant growth parameters

Shoot dry mass (SDM) and root dry mass (RDM) was determined after drying to mass constancy at 65°C with 15% relative humidity. The deepest root penetration visible along the observation window was recorded as rooting depth (RD). The WinRHIZO root analysis system (Regent Instruments, Quebec, Canada) was used to measure total root length (TRL) and fine root length (FRL; 0–0.2 mm diameter) of excavated roots by optical scanning. Root hair size (RHS) was determined after magnification of high-resolution digital photographs taken from roots growing along the observation windows (Zeiss Axiovision software, Oberkochen, Germany).

### Plant nutrient analysis

Dried leaves were ground and 200–500 mg material was subjected to microwave digestion (Mars 6, CEM, Charlotte, USA) with 5 ml HNO_3_ (65%) and 3 ml H_2_O_2_ (30%) for 25 min at 210°C. Calcium (Ca), potassium (K), phosphorus (P), magnesium (Mg), sulphur (S), iron (Fe), manganese (Mn), copper (Cu), and zinc (Zn) concentrations were determined via inductively coupled plasma optical emission spectrometry (ICP-OES, Thermo Fisher Scientific, Waltham, USA); total carbon (C) and N were determined via elemental analysis (Elementary Vario El cube, Elementar, Langenselbold, Germany).

### RNA extraction and plant gene expression analysis

Leaves were retrieved with sterile forceps, excess RNAlater solution was removed and samples were immediately submerged in liquid nitrogen and pulverized. The RNeasy Plant Mini Kit (Qiagen GmbH, Hilden, Germany) was used to extract RNA from 100 mg leaves. After quantification of RNA by NanoDrop spectrophotometer (Thermo Fisher Scientific), cDNA was synthesized from 2 μg of RNA with the High-Capacity cDNA Reverse Transcription Kit with RNase Inhibitor (Applied Biosystems, Foster City, USA).

Based on an extensive literature search, 30 target genes from wheat (*T. aestivum*) were selected for expression analyses (Table S1, Supporting Information). These genes are associated with biotic and abiotic stress responses [e.g. genes involved in signaling-pathways mediated by salicylic acid (SA), jasmonic acid (JA), and ethylene] and in the first line of defense (MAP kinases, peroxidase, catalase, and superoxide dismutase). In addition, several genes involved in N-metabolism and Fe-transport in wheat were analyzed. The reference genes coding for ubiquitin (Ubi) and elongation factor 1α (EF1α) were used for normalization. The comparative ΔΔCT method (Livak and Schmittgen 2001) was applied. The target and endogenous control genes were validated and only primers with 100% (± 10%) efficiency were used. For this purpose, the target gene primers and the endogenous controls were used to amplify RNA (cDNA) from winter wheat with a concentration range of 100 ng to 100 pg in a series of 10-fold dilutions in triplicate. The qPCR was performed with Power SYBR Green Supermix using a peqSTAR 96Q thermal cycler (PEQLAB Biotechnologie GmbH, Erlangen, Germany) as described previously (Chowdhury et al. 2019). A total of 28 primer pairs, that met the criteria, were selected for further analyses (Table S1, Supporting Information). QPCR of four biological replicates was performed in three technical replicates (Chowdhury et al. 2019). Data were first normalized to the endogenous control and logarithmically transformed to fold change differences. The standard error of the mean was calculated from the average of the biological replicates.

### Analysis of plant hormones and stress-related metabolites

The plant hormones (JA and SA) and selected stress metabolites were analyzed in homogenized and shock-frozen plant tissue. UHPLC-MS analysis of phytohormones in shoots was carried out as described by Moradtalab et al. (2020). Total phenolics were determined, after extraction with 80% v/v methanol, spectrophotometrically at 750 nm, using the Folin method (Swain and Hillis 1959). Proline analysis was conducted spectrophotometrically at 520 nm after acetic acid and acid ninhydrin derivatization (Moradtalab et al. 2018). The 1,1-diphenyl-2-picrylhydrazyl radical method was used to evaluate the free radical scavenging activity of total antioxidants (T-AO; Moradtalab et al. 2020). Ascorbate peroxidase (APX, EC 1.11.1.11) activity was recorded by spectrophotometric method (Boominathan and Doran 2002). A Spectrophotometer U-3300 (Hitachi, Tokyo, Japan) was used for all spectrophotometric determinations.

### Analysis of root exudates and RH metabolites

HPLC-profiling of organic acids, sugars, and amino acids in the RH soil solutions in the 80% methanol extracts of the sorption filters was conducted as described by Windisch et al. (2021). For phenolic compounds and BXs, identification was performed with positive/negative switching LC-MS on a QExactive Plus Electrospray Mass Spectrometer (Thermo Fisher Scientific) coupled to an Agilent 1290 Ultra Performance Liquid Chromatography System (Table S2, Supporting Information). Quantitative analysis of identified compounds was conducted by comparison with known standards using a Shimadzu LC10 HPLC system (Table S2,Supporting Information).

### Microbial community DNA extraction and amplicon sequencing

TC-DNA was extracted from complete RH pellets or 0.5 g RA soils (wet weight) using FastPrep FP24 bead-beating system (twice, each 30 s, 5.5 m s^−1^) and FastDNA Spin Kit for soil (MP Biomedicals, Santa Ana, USA) according to manufacturer’s recommendations. Extracted TC-DNAs were purified using GeneClean Spin Kit (MP Biomedicals). TC-DNA yield and quality was checked by agarose gel electrophoresis and stored at −20°C.

For bacterial community profiling based on the V3–V4 region of the 16S rRNA gene (16S), PCR was performed as described in Babin et al. (2021) with primers 341F/806R. Adding of Illumina sequencing adapters and sample-specific dual indexes, and preparation of libraries were done as described in Fernandez-Gnecco et al. (2022). Sequencing was carried out on an Illumina MiSeq platform using Reagent Kit v2 (2 × 250 bp; Illumina, San Diego, USA) following manufacturer’s instructions. 16S reads were processed into amplicon sequence variants (ASVs) using DADA2 version 1.10.0 (Callahan et al. 2016), which were taxonomically annotated with SILVA SSU rel. 132 database (Quast et al. 2013) as described in more detail in Fernandez-Gnecco et al. (2022). ASVs with less than five reads across the full data set were excluded from analysis. Furthermore, ASVs classified as chloroplasts, mitochondria (both < 0.3% of total reads) or present in the negative control were discarded. This resulted in a final number of 7811 ASVs and on average 28 711 quality reads per sample, which was sufficient to cover the diversity in all samples (Figure S3A, Supporting Information).

Profiling of fungal communities was carried out based on high-throughput amplicon sequencing of the Internal Transcribed Spacer (ITS2) region using a sample-specific barcoded primer pair (ITS86F/ITS4; White et al. 1990, Op de Beeck et al. 2014; Table S3, Supporting Information) as previously described (Sommermann et al. 2018, Babin et al. 2021). In brief, the amplification was performed with three PCRs per sample (10 ng TC-DNA each) at different annealing temperatures (56°C  ± 2°C) using the Q5 High-Fidelity 2x Master Mix (New England Biolabs, Ipswich, USA) at 25 cycles. Subsequently, technical replicates were mixed and purified by MinElute PCR Purification Kit (Qiagen) with a final elution volume of 12 μl 10 mM Tris-HCl (pH 8.5). The concentration of each sample was checked by a Qubit ® 3.0 Fluorometer (Invitrogen, Carlsbad, USA) and mixed equimolarly. ITS2 sequencing was conducted as previously described (Babin et al. 2021) on the Illumina MiSeq platform in paired-end mode (2 × 300 bp). Different trimming steps (barcodes, primers, and adapters) including the FASTX toolkit5 and raw sequence merging using FLASH (Magoč and Salzberg 2011) were conducted. With a local GALAXY Bioinformatics Platform in combination with the fungal UNITE database v8.0 (UNITE Community 2019), the database-dependent strategy (Antweiler et al. 2017) was performed. For this, sequences of all samples were aligned with the database (e-value ≤ 0.001) keeping only results fulfilling the following conditions: min. alignment length ≥ 200 bp and min. similarity ≥ 97%. To generate the ASV abundance table, the SH-numbers of the UNITE database were used as identifiers counting the sequences per assignment. Finally, a total of 1361 ASVs could be retrieved with an average of 86 958 reads per sample (Figure S3B, Supporting Information).

### Shotgun metagenome sequencing

In order to profile the functional potential of the RH microbiome, shotgun metagenomic libraries of TC-DNA were prepared using NEBNext Ultra II FS DNA Library Prep Kit (New England Biolabs) according to the manufacturer’s protocol with the following modifications. Enzymatic shearing was performed using 100 ng DNA of each sample and an optimized incubation time of 15 min. The NEBNext Adaptor for Illumina was diluted (1:10) and no size selection was performed on the adaptor ligated DNA. The purified samples were amplified for ten cycles using the i7 and i5 primers of the NEBNext Multiplex Oligos for Illumina (Dual Index Primers Set 1) (New England Biolabs). The final products were purified by MagSi-NGSPrep Plus kit (Steinbrenner, Wiesenbach, Germany) twice using two different library-to-bead ratios (0.6 and 0.8, respectively). The size distribution and concentration of the libraries were evaluated on a Fragment Analyzer Automated CE System (Advanced Analytical Technologies, Orangeburg, USA) using the DNF-474 High Sensitivity NGS Fragment Analysis Kit (1–6000 bp). The libraries were diluted to 1 nM and sequenced on a NextSeq550 sequencer (Illumina) using the NextSeq 500/550 High Output Kit v2.5 (300 cycles) after equimolar pooling.

The shotgun metagenome sequencing produced a high-quality output with a sequencing depth of 16 million reads per sample. Raw reads obtained from NextSeq sequencing were processed on a GALAXY Bioinformatics platform. They were trimmed and Illumina adaptor sequences were removed using TrimGalore (Galaxy Version 0.6.3; Krueger et al. 2021) with min. read length 50 bp and min. Phred quality = 20. For the read-based analysis, forward and reverse reads were merged using Illumina paired-end read merger (PEAR Galaxy Version 0.9.6.1; Zhang et al. 2014). PhiX contamination was removed using bbduk (version 38.93; Bushnell et al. 2017) with kmer length = 31. Trimming and merging of reads resulted in 178 750 308 reads covering 91.7% of all sequences. Coverage of the metagenomic data set was estimated using Nonpareil (Galaxy Version 3.1.1.0; Rodriguez-R and Konstantinidis 2014) in alignment mode. The coverage was 7%–10%, which indicates a read-based analysis (Figure S7, Supporting Information).

Preprocessed reads were taxonomically classified using the MGX pipeline [Kraken with customized Prokaryotic protein database plus DIAMOND with the National Center for Biotechnology Information’s nonredundant (NCBI-nr) protein database] implemented on the MGX platform (Jaenicke et al. 2018) with default parameter (e = 10^−5^, identity = 80%). 46.8% of reads were classified as bacteria and considered for further analysis. Only a small fraction of archaea (0.52%) and fungi (0.047%) could be classified, which were not enough to be representative and were therefore not further analyzed.

To analyze potential plant-beneficial functions, a customized database was established. Functions were selected based on a prescreening using the online tool for metagenome analysis MG-RAST (Keegan et al. 2016). Protein sequences of functions, which could be relevant for plant–microbe interactions with potential direct (e.g. nitrogen fixation) or indirect (e.g. antimicrobial compounds) beneficial effects for plant growth and health (Table S4, Supporting Information), were extracted based on KO numbers from the latest version of the KEGG database (November 2022) using a customized R-Script and stored in the customized database. The database was extended for additional potential plant-beneficial functions described in literature (Glick 2012, Kuzmanović et al. 2018, Cania et al. 2019) and downloaded from KEGG database (November 2022) (Table S4, Supporting Information). Preprocessed reads were run against the customized database using DIAMOND (Version 2.0.15; Buchfink et al. 2015) with alignment mode = blastx, more-sensitive mode, e = 10^−5^, identity = 80% and query coverage = 50%.

For linking taxa and functions, the fastq-sequences of significantly differential abundant functions were extracted using seqtk_subseq (Galaxy Version 1.3.1; Li 2012) from the preprocessed sequences and taxonomically classified on the MGX platform as described above.

### Data analyses and statistics

Statistical analyses were performed in R (R Core Team 2019) including the packages *agricolae* (De Mendiburu 2017), *vegan* (Oksanen et al. 2019), phyloseq (McMurdie and Holmes 2013), *RColorBrewer* (Neuwirth 2014), *data.table* (Dowle and Srinivasan 2023), *ggplot2* (Wickham 2016), *pheatmap* (Kolde 2019), *dplyr* (Wickham et al. 2019), *MASS* (Venables and Ripley 2002), *rcompanion* (Mangiafico 2020), *car* (Fox et al. 2020), *emmeans* (Lenth 2020), *stats* (R Core Team 2019), *ape* (Paradis and Schliep 2019), *writexl* (Ooms 2023), *gplots* (Warnes et al. 2022), *rioja* (Juggins 2023), *edgeR* (Robinson et al. 2010), *ARTool* package (Kay et al. 2021), *ComplexHeatmap* (Gu et al. 2016), and *mvabund* (Wang et al. 2012).

ITS/16S: the effect of tillage practice and N-fertilization intensity on microbial community composition was tested by PERMANOVA (10 000 permutations) based on Bray–Curtis dissimilarity matrix (ITS: count data; 16S: log relative abundance) and nonmetric multidimensional scaling (NMDS) was used for ordination. Alpha-diversity (species richness, Pielou, Shannon) was calculated by 100 times random subsampling to the lowest number of reads (ITS: 51 670; 16S: 18 544). The effects of tillage practice and N-fertilization intensity on alpha-diversity were tested by two way-ANOVA followed by *post hoc* Tukey’s HSD test (*P* < .05). If ANOVA assumptions failed, data were transformed by Tukey’s Ladder of Power. Heatmaps represent the 30 most abundant microbial genera based on relative abundances (Euclidean distances). Differences in relative abundances in the RH on ASV level were tested by likelihood ratio tests under negative binomial distribution and generalized linear models (edgeR) for tillage practice (MP vs. CT) and N-fertilization intensity (Int vs. Ext) following developers’ recommendation (filter criterion: ASV present in min. three samples; each comparison group *n* = 8).

Metagenome: low abundance features were removed by filtering the data for at least two hits in two samples. Data were analyzed based on relative abundance of reads by dividing the classified reads by the total number of reads per sample. Results were converted into percentages by multiplying with 100. PERMANOVA analysis (Bray–Curtis dissimilarity, 10 000 permutations) was performed to detect effects of tillage practice, N-fertilization intensity and their possible impact on microbial community and functional composition. Visualization of dissimilarities between samples was carried out with NMDS using the Bray–Curtis distance matrix. Differential analysis was performed with edgeR (Chen et al. 2016) filtered for at least one hit in three samples.

Plant: the effects of tillage practice and N-fertilization intensity on the metabolic profiles of shoots, RH soil solutions and RA soils, as well as on the nutrient status of the plants were analyzed by means of a two-way ANOVA. The assumptions of homogeneity of variance and normality of the residuals were visually checked with the *performance* package (Lüdecke et al. 2021). If the conditions for ANOVA failed, the data were Tukey-transformed and checked again. If the requirements were still not met, an ART-ANOVA was performed. A pairwise mean comparison was performed on the basis of *P* ≤ .05 using Tukey-HSD test. PERMANOVA (10 000 permutations) analyses were performed based on Bray–Curtis dissimilarities calculated from ΔCt values of 28 genes and 15 LMW organic compounds and secondary metabolites of the RH soil solution and principal coordinates analysis (PCoA).

Correlation analysis: Kendall rank correlations of metabolites of the apical RH soil solution against ASVs were carried out using the kendalltau function (Knight 1966) of the python package *scipy* (Virtanen et al. 2020). To reduce the number of calculations only metabolites with significant differences between the treatments and tillage responders (edgeR; relative abundance > 0.5%) were considered. Correlations with Kendall’s tau ≥ 0.5 or ≤ −0.5 with *P* < .01 were considered as significantly correlated and visualized in a correlation matrix.

## Results

### Plant biomass, root growth characteristics, nutritional status, and grain yield

Winter wheat grown under Ext exhibited a significantly higher RDM than under Int (Table 1). TRL and SDM were lowest under Int, regardless of the tillage practice. Overall, Ext significantly increased RHS and RD over Int, while CT significantly increased FRL over MP (Table 1). Final grain yields determined for the whole field experiment ranged between 6.3 and 6.6 t ha^−1^ without significant differences among agricultural practices (data not shown).

**Table 1.** Effect of long-term agricultural practice on shoot dry mass (SDM), root dry mass (RDM), total root length (TRL), fine root length (FRL), root hair size (RHS), and rooting depth (RD) of winter wheat (cv. Lemmy; EC 59). MP – mouldboard plough, CT – cultivator tillage, Ext – extensive N-fertilization intensity without fungicides and growth regulators, and Int – intensive N-fertilization intensity with pesticides and growth regulators. SDM, RDM, and TRL data are adjusted to 10 tillers, which represents the average number of tillers in one plant. Values are presented as means ± standard deviation of four replicates. Means not sharing any letters are significantly different by the Tukey-test (*P* ≤ .05).

	MP-Int	MP-Ext	CT-Int	CT-Ext
SDM (g)	15.7 ± 2.2 a	21.3 ± 2.9 a	19.9 ± 5.1 a	21.8 ± 1.0 a
RDM [g)	2.44 ± 0.36 c	3.71 ± 0.34 ab	2.7 ± 0.72 bc	3.89 ± 0.44 a
TRL (m)	8.2 ± 2.5 b	18.0 ± 5.1 ab	15.6 ± 6.0 ab	24.1 ± 8.1 a
FRL (cm)	239.1 ± 17.0 b	254.6 ± 12.8 b	358.6 ± 33.8 a	355.6 ± 9.3 a
RHS (mm)	0.44 ± 0.03 ab	0.57 ± 0.09 a	0.41 ± 0.06 b	0.56 ± 0.06 a
RD (cm)	38.9 ± 4.3 b	55.8 ± 5.0 a	39.4 ± 4.7 b	55.7 ± 4.6 a

Elemental concentrations in flag leaves were affected by N-fertilization intensity. Plants grown in CT-Ext soil exhibited a significantly lower N, Mg, Ca, and S concentration in the leaf tissue compared to the other treatments. CT-Ext plants had the lowest C and K concentrations in the leaf tissue. Only marginal differences were observed for leaf micronutrient contents, with all above the deficiency threshold (Table 2; Table S5, Supporting Information). Only K levels were below the deficiency threshold (Campbell 2000) across all treatments (Table 2).

**Table 2.** Impact of long-term agricultural practice on the nutrient status of winter wheat (cv. Lemmy; EC 59). MP – mouldboard plough tillage, CT – cultivator tillage, Ext – extensive N-fertilization intensity without fungicides and growth regulators, and Int – intensive N-fertilization intensity with pesticides and growth regulators, DM- dry mass. Values are presented as means ± standard deviation of four replicates. Means not sharing any letters are significantly different by the Tukey-test (*P* ≤ .05).

	DT^#^	MP-Int	MP-Ext	CT-Int	CT-Ext
Macronutrients (g kg^−1^ shoot DM)					
C_total_	–	449.9 ± 2.3 ab	450.4 ± 1.9 ab	453.8 ± 2.0 a	448.8 ± 2.5 b
N_total_	30.0	37.1 ± 1.0 a	35.88 ± 0.3 ab	37.9 ± 2.3 a	31.4 ± 3.5 b
P	1.5	2.1 ± 0.1 a	2.54 ± 0.3 a	2.4 ± 0.3 a	2.1 ± 0.3 a
K	20.0	18.4 ± 1.8 ab	17.25 ± 0.2 ab	19.1 ± 2.2 a	15.7 ± 1.1 b
Mg	1.0	1.8 ± 0.1 a	1.63 ± 0.1 ab	1.73 ± 0.2 a	1.4 ± 0.1 b
Ca	1.5	6.6 ± 0.3 a	5.69 ± 0.2 bc	5.9 ± 0.3 b	5.3 ± 0.3 c
S	1.0	4.6 ± 0.1 a	3.44 ± 0.1 b	3.9 ± 0.3 b	2.9 ± 0.2 c
Micronutrients (mg kg^−1^ shoot DM)					
Cu	3.0	4.6 ± 0.2 ab	4.8 ± 0.2 a	4.0 ± 0.5 b	4.2 ± 0.2 ab
Fe	25.0	51.4 ± 24.0 a	66.6 ± 25.9 a	67.6 ± 10.4 a	100.9 ± 30.2 a
Mn	15.0	52.6 ± 1.4 a	52.9 ± 5.5 a	57.1 ± 10.1 a	55.3 ± 4.3 a
Zn	15.0	14.9 ± 0.7 a	15.8 ± 0.9 a	15.4 ± 3.0 a	16.6 ± 3.2 a

# Deficiency threshold (DT) of macro- and micronutrients after Campbell (2000).

No symptoms of leaf rust, but symptoms of powdery mildew caused by *Blumeria graminis* were visible on the lower leaves and stem. Although severity of the infestation was not exactly quantified, a stronger visible infestation in MP and no or only weak infestation in CT treatments was observed.

### Stress-related metabolites and gene expression in wheat leaves

The tillage practice was identified as a dominant factor determining metabolic stress adaptations (Table 3; Table S6, Supporting Information). Lower concentrations of stress hormones (JA and SA) and metabolic stress indicators (T-AO, proline, and APX) were detected in leaves of plants under CT (Table 3). N-fertilization intensity influenced stress-related metabolites, especially in the CT treatments. Significantly lower concentrations of stress hormones and proline were detected in CT-Int compared to CT-Ext plants (Table 3; Table S6, Supporting Information).

**Table 3.** Phytohormones (SA = salicylic acid and JA = jasmonic acid) and stress-associated metabolites (T-AO = total antioxidants and APX = ascorbate peroxidase) in leaves of winter wheat (cv. Lemmy, EC 59) grown under different long-term agricultural practices. MP – mouldboard plough tillage, CT – cultivator tillage, Ext – extensive N-fertilization intensity without fungicides and growth regulators, and Int – intensive N-fertilization intensity with pesticides and growth regulators. Values are presented as means ± standard deviation of four replicates. Means not sharing any letters are significantly different by the Tukey-test (*P* ≤ .05). FW – fresh weight.

	MP-Int	MP-Ext	CT-Int	CT-Ext
Stress-associated phytohormones (ng g^−1^ FW)				
SA	0.58 ± 0.04 a	0.63 ± 0.04 a	0.29 ± 0.02 c	0.47 ± 0.04 b
JA	0.51 ± 0.03 a	0.54 ± 0.04 a	0.23 ± 0.01 c	0.41 ± 0.03 b
Stress indicators (Unit g^−1^ FW)				
Proline	2.6 ± 0.2 b	3.3 ± 0.2 a	1.6 ± 0.1 d	2.1 ± 0.1 c
Phenolics	5.0 ± 0.3 a	5.2 ± 0.3 a	5.2 ± 0.2 a	3.6 ± 0.2 b
T-AO	87.4 ± 4.0 a	88.4 ± 3.6 a	55.6 ± 5.8 b	61.1 ± 5.7 b
APX	599.9 ± 39.1 a	618.2 ± 40.2 a	383.9 ± 24.9 b	415.8 ± 27.2 b

**Figure 1.**
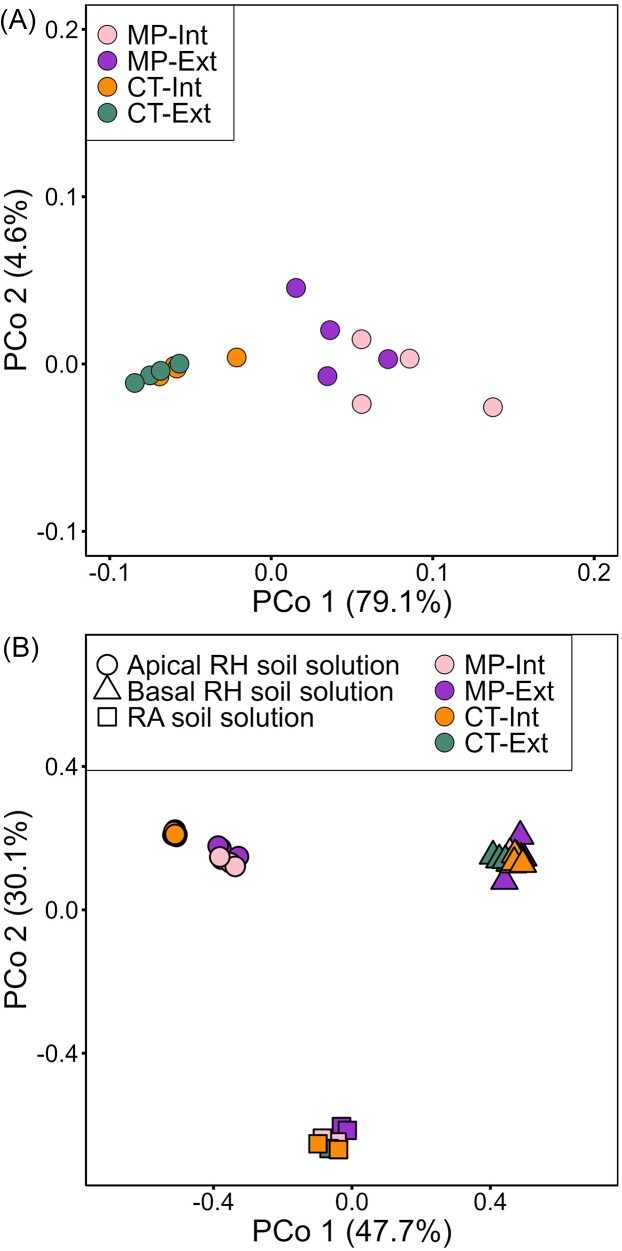
PCoA based on (A) the expression of 28 selected genes (ΔCt values) and (B) of 15 low molecular weight (LMW) organic compounds and secondary metabolites of the apical and basal rhizosphere (RH) soil solution as well as the root-affected (RA) soil solution of winter wheat (cv. Lemmy, EC 59) grown in different long-term agricultural practices (MP – mouldboard plough, CT – cultivator tillage, Ext – extensive N-fertilization intensity without fungicides and growth regulators, and Int – intensive N-fertilization intensity with pesticides and growth regulators) of the long-term experiment in Bernburg.

The influence of the different agricultural practices on the expression levels of 28 selected genes involved in stress responses was analyzed by qPCR in leaves. In line with PERMANOVA analysis (Table 4), PCoA showed that samples clustered according to tillage practice with minor differences between N-fertilization intensities (Fig. 1A). Pairwise comparison of gene expression revealed that 21 out of 28 genes showed significantly enhanced expression in plants from MP compared to CT soils in both Int and Ext fertilization (Figure S4, Supporting Information). Nitrate reductase 1 (*TaNR1*) and nitrite reductase (*TaNIR*) were identified as significantly increased in Int compared to Ext treatment in both CT and MP tillage practice, according to Tukey’s HSD analyses (data not shown).

**Table 4.** Effect of long-term agricultural practices on plant gene expression in leaves of wheat plants (cv. Lemmy, EC 59) as well as on taxonomic and functional composition of microbial communities expressed as explained variance (EV). Gene expression is based on 28 selected genes expressed in leaves. Taxonomic composition of microbial communities is based on 16S rRNA gene or ITS amplicon sequencing in the root-affected (RA) soil and rhizosphere (RH). Functional composition is based on metagenome sequencing of RH and analyzed using a customized database for plant-beneficial bacterial functions (*n* = 321; filtered two hits, two samples, no log transformation). Analysis is based on PERMANOVA using Bray–Curtis distances (10 000 permutations). Significant effects (*P*-value = *P*) are marked with * *P* < .05, ** *P* < .01, and *** *P* < .001. ns – not significant.

	Gene expression		16S RA soil		16S RH		ITS RA soil		ITS RH		RH metagenome	
Factor	EV (%)	*P*	EV (%)	*P*	EV (%)	*P*	EV (%)	*P*	EV (%)	*P*	EV (%)	*P*
Tillage	70.9	***	17.8	***	29.2	***	28.2	***	23.6	***	31.6	***
N-Fertilization	6.8	*	9.6	*	8.2	ns	15.7	**	17.1	***	5.5	ns
Tillage:N-Fertilization	2.3	ns	7.7	ns	6.7	ns	4.6	ns	12.5	***	25.3	***
Residuals	20.0		64.9		55.8		51.5		46.8		37.4	

### RH metabolites

PCoA analysis showed that the metabolite profiles differed strongly among root zones (Fig. 1B). The level of all measured metabolites was highest in the apical root zone (Tables S6 and S7, Supporting Information) and differentiation of samples according to tillage practice was visible (Fig. 1B). Therefore, the apical root zone was investigated in more detail to elaborate, which metabolites contributed to the differentiation.

CT treatments had higher levels of specific metabolites like succinic acid, asparagine, tryptophan, and trehalose, compared to MP (Table 5; Table S6, Supporting Information). Various secondary metabolites including benzoic acid, flavonoids (quercetin and naringenin), phenolic acids (caffeic acid, cinnamic acid, and para-coumaric acid), and a range of BXs were detected. For BXs solely metabolites of the unstable BX DIMBOA were detectable in the RH soil solution, such as MBOA and 6-Methyl-2H-1,4-benzoxazin-3(4H)-one (MeBOA). Tillage practice significantly affected the composition and quantity of the BXs profiles. A higher MBOA concentration was detected in the apical zones of CT treatments, while MeBOA and benzoic acid were significantly lower compared to MP treatments (Table 5; Table S6, Supporting Information). Apical root zones of plants grown in MP-Int exhibited the highest concentrations of MeBOA and benzoic acid. In the RH soil solution collected from older, basal root zones, secondary metabolites tended to be higher in CT treatments (Table 5).

**Table 5.** Effect of long-term agricultural practices on primary and secondary metabolites in RH soil solution of apical and basal roots of winter wheat (cv. Lemmy; EC 59). MP – mouldboard plough tillage, CT – cultivator tillage, Ext – extensive N-fertilization intensity without fungicides and growth regulators, and Int – intensive N-fertilization intensity with pesticides and growth regulators. Values are presented as means ± standard deviation of four replicates. Means not sharing any letters are significantly different by the Tukey-test (*P* ≤ .05).

	Metabolites (nmol cm^−1^ root length)			
	MP-Int	MP-Ext	CT-Int	CT-Ext
Apical RH soil solution				
Asparagine	3.74 ± 0.21 b	3.82 ± 0.13 b	6.31 ± 0.14 a	6.24 ± 0.08 a
Benzoic acid	10.4 ± 1.17 a	7.98 ± 0.93 b	5.58 ± 0.66 c	5.29 ± 0.39 c
Caffeic acid	6.7 ± 0.37 a	5.78 ± 0.51 b	6.28 ± 0.40 ab	6.17 ± 0.33 ab
Cinnamic acid^#^	8.84 ± 1.13 a	7.63 ± 0.59 b	7.98 ± 0.50 ab	7.86 ± 0.35 ab
MBOA^##^	0.71 ± 0.12 b	0.29 ± 0.29 b	35.8 ± 5.99 a	41.63 ± 4.05 a
MeBOA	10.66 ± 0.75 a	9.13 ± 0.62 b	3.43 ± 1.00 c	2.67 ± 0.04 c
Succinic acid	1.11 ± 0.14 b	1.33 ± 0.13 b	1.88 ± 0.22 a	1.82 ± 0.22 a
Trehalose	16.00 ± 4.32 c	17.75 ± 6.24 c	57.50 ± 11.86 b	80.00 ± 9.80 a
Tryptophan	0.009 ± 0.001 b	0.010 ± 0.001 b	0.015 ± 0.001 a	0.015 ± 0.001 a
Basal RH soil solution				
Benzoic acid	2.03 ± 1.39 ab	1.63 ± 1.29 b	4.09 ± 1.02 a	2.42 ± 0.74 ab
Caffeic acid	0.66 ± 0.45 b	0.97 ± 0.26 ab	1.31 ± 0.11 a	1.06 ± 0.14 ab
Cinnamic acid	0.97 ± 0.12 bc	0.95 ± 0.08 c	1.24 ± 0.04 a	1.16 ± 0.11 ab
p-Coumaric acid	0.74 ± 0.10 b	0.74 ± 0.06 b	0.97 ± 0.04 a	0.91 ± 0.09 a

# Tukey transformed data.

## Art-ANOVA.

### Phylogenetic composition of the microbiome

The microhabitat (RA soil or RH) was identified as the driving factor for the bacterial/archaeal and fungal beta-diversity (*R*^2^ = 27% and *R*^2^ = 40.5%, respectively; both *P* < .001; PERMANOVA). This was also apparent at the level of the major abundant genera (Figure S5A, Supporting Information). Typical bacterial/archaeal genera detected in RA soil had the closest affiliation to *Nitrososphaeraceae*, Acidobacteria subgroup 6 and RB41. In contrast, *Bacillus, Devosia, Pedobacter*, and *Massilia* were more prevalent in the RH (Figure S5A, Supporting Information). For fungi, the genera *Mortierella, Chaetomium, Fusarium, Gibellulopsis*, and *Solicoccozyma* dominated the RA soil while in the RH *Zymoseptoria, Pseudogymnoascus, Mycosphaerella*, and *Filobasidium* prevailed (Figure S5B, Supporting Information). In addition, effects of tillage practice were apparent, especially in RH on the level of the most abundant fungal genera such as *Rhizopu*s and *Trichoderma* or *Mortierella* and *Chrysosporium* enriched in CT or MP treatments, respectively (Figure S5, Supporting Information).

Bacterial and archaeal alpha-diversity was significantly affected by N-fertilization intensity (Shannon, Richness) or tillage and tillage x N-fertilization (Pielou) in RA soil. While Pielou indices were higher in RA soil under CT than under MP, richness and Shannon indices were highest in RA soil under Ext fertilization independent of the tillage practice (Table S8, Supporting Information). No significant effects of the agricultural practice on bacterial and archaeal alpha-diversity were observed in the RH (Table S8, Supporting Information). Similarly, for fungi only a significantly higher richness in Ext compared to Int treatments was observed in RH. However, in RA, CT-Ext soils exhibited a significantly higher fungal richness or Shannon diversity compared to MP-Int or CT-Int, respectively (Table S9, Supporting Information).

In both RA soil and RH, tillage practice exhibited a stronger effect on the microbial community composition than N-fertilization intensity (PERMANOVA, Table 4). The effect of N-fertilization intensity was stronger for fungi than for bacteria. A significant interaction effect between both agricultural practices was found on the fungal RH communities (Table 4).

**Figure 2.**
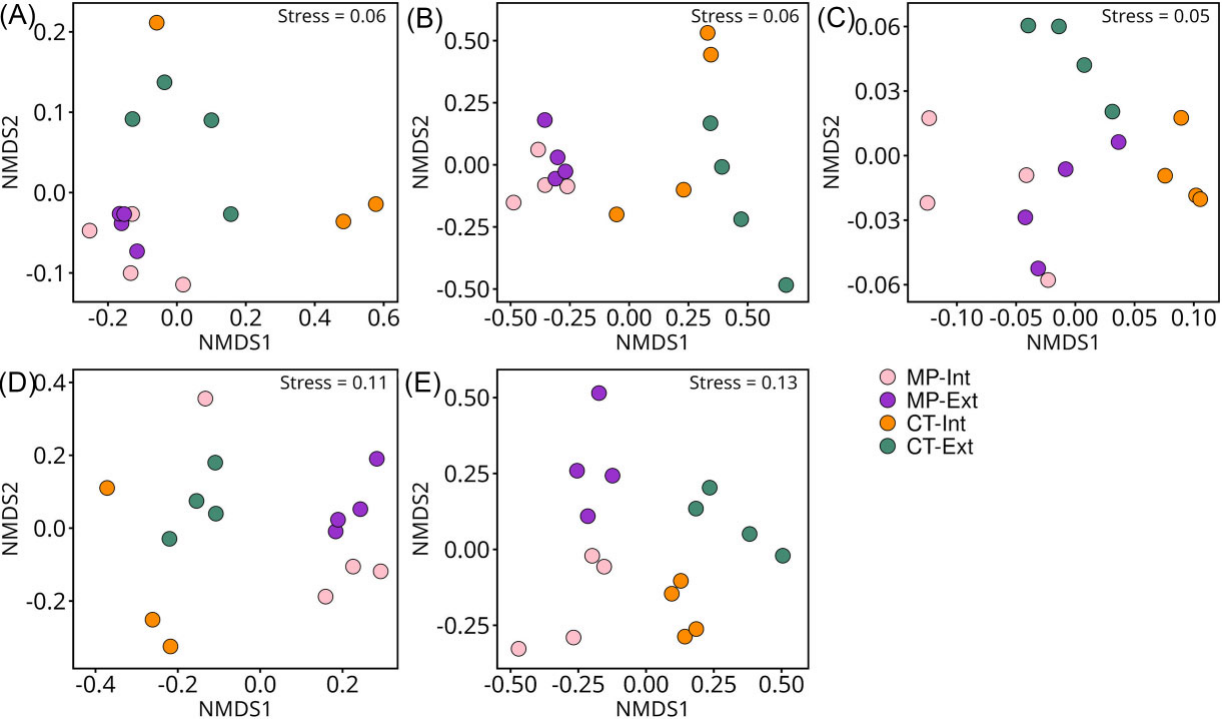
NMDS analysis showing the effects of agricultural practice (MP – mouldboard plough; CT – cultivator tillage; Int – intensive N-fertilization intensity with pesticides and growth regulators; and Ext – extensive N-fertilization intensity without fungicides and growth regulators) in the long-term field experiment Bernburg on (A) root-affected (RA) soil, (B) rhizosphere (RH) archaeal/bacterial community structure, (C) RH potential plant-beneficial functional composition of the bacterial community (shotgun metagenomic sequences annotated with a customized database; Table S4, Supporting Information), and (D) RA soil and (E) RH fungal community structure of winter wheat (cv. Lemmy, EC 59). Stress indicates the ordination stress value of each individual NMDS analysis.

The NMDS ordination of microbial communities substantiated the results of the PERMANOVA showing a distinct separation between CT vs. MP. For archaea/bacteria, CT samples displayed a higher variability among replicates than MP. In contrast to archaea/bacteria, an additional clustering based on N-fertilization intensity was observed for fungal communities in both microhabitats (Fig. 2A, B, D, and E).

Since the RH microbiome has important impacts on plant development and health, differentially abundant taxa (ASVs) among tillage practices or N-fertilization intensity were identified. Many of the bacterial and archaeal taxa that were significantly increased in relative abundance in the RH of MP in contrast to CT treatment belonged to Actinobacteria and Acidobacteria e.g. sequences with closest affiliation to *Micrococcaceae, Agromyces*, RB41, Acidobacteria Subgroup 6, but also to *Terrimonas, Nitrospira*, and *Nitrososphaeraceae* (Table 6; Table S10, Supporting Information). Taxa with higher relative abundance in CT compared to MP belonged to *Bacillus, Sphingomonas, Devosia, Pseudoxanthomonas*, and *Saccharimonadaceae* (Patescibacteria) (Table 6; Table S10, Supporting Information). No bacterial/archaeal ASVs were found that responded to either Int or Ext in the RH (FDR < 0.05).

**Table 6.** Bacterial and archaeal species (ASV) with differential relative abundance in the rhizosphere (RH) of winter wheat (cv. Lemmy, EC 59) grown in mouldboard plough (MP) or cultivator tillage (CT) soils (FDR < 0.05). Only discriminative species with mean > 0.5% are displayed. Significantly enriched taxa are marked in bold. Mean ± standard deviation is shown (*n* = 8).

Phylum	Genus	Species	ASV	MP	CT
Acidobacteria	*RB41*	*RB41_unclassified*	ASV4136	**0.5 ± 0.2**	0 ± 0.1
Actinobacteria	*Micrococcaceae_unclassified*	*Micrococcaceae_unclassified*	ASV49496	**2.3 ± 0.8**	0.9 ± 0.7
Actinobacteria	*Agromyces*	*Agromyces_unclassified*	ASV660	**0.7 ± 0.1**	0.2 ± 0.2
Actinobacteria	*Micrococcaceae_unclassified*	*Micrococcaceae_unclassified*	ASV16162	**0.5 ± 0.2**	0 ± 0.1
Bacteroidetes	*Terrimonas*	*Terrimonas_unclassified*	ASV11972	**0.7 ± 0.2**	0.4 ± 0.2
Nitrospirae	*Nitrospira*	*uncultured soil bacterium*	ASV33194	**0.6 ± 0.1**	0.2 ± 0.2
Nitrospirae	*Nitrospira*	*Nitrospira_unclassified*	ASV35563	**0.5 ± 0.1**	0 ± 0.1
Thaumarchaeota	*Nitrososphaeraceae_unclassified*	*Nitrososphaeraceae_unclassified*	ASV42131	**1.4 ± 0.5**	1.0 ± 0.3
Firmicutes	*Bacillus*	*Bacillus_unclassified*	ASV47072	0.2 ± 0.1	**1.0 ± 0.4**
Firmicutes	*Bacillus*	*Bacillus_unclassified*	ASV10264	0.2 ± 0.1	**0.7 ± 0.3**
Patescibacteria	*Saccharimonadaceae_unclassified*	*Saccharimonadaceae_unclassified*	ASV51676	0 ± 0	**0.5 ± 0.9**
Proteobacteria	*Sphingomonas*	*Sphingomonas_unclassified*	ASV40734	0.2 ± 0.1	**0.6 ± 0.2**
Proteobacteria	*Devosia*	*Devosia_unclassified*	ASV1636	0.2 ± 0.1	**0.7 ± 0.3**
Proteobacteria	*Pseudoxanthomonas*	*Pseudoxanthomonas_unclassified*	ASV48785	1.8 ± 0.3	**3.2 ± 1.8**
Thaumarchaeota	*Nitrososphaeraceae_unclassified*	*Nitrososphaeraceae_unclassified*	ASV24360	0.1 ± 0	**0.6 ± 0.2**

Most of the fungal representatives, which showed a significantly higher relative abundance in CT, belonged to the phylum Ascomycota and the class Sordariomycetes, like the species with the closest affiliation to *Chaetomium* sp., *Trichoderma petersenii*, and *T. piluliferum, Fusarium* sp., and *Stachybotrys chartarum* (Table 7; Table S11, Supporting Information). Additionally, species with closest sequence similarity to *Penicillium aethiopicum, Articulospora* sp., *Tausonia pullulans*, and *Rhizopus arrhizus* were indicative for CT. In contrast, the identified responders in MP belonged to different phyla like Ascomycota (*Ascomycota* sp., *Chrysosporium lobatum, Pseudogymnoascus pannorum*, and *Acremonium persicinum*), Mortierellomycota (*Mortierella fimbricystis* and *M. antarctica*), Basidiomycota families (*Sporidiobolaceae* sp. and *Strophariaceae* sp.), and Olpidiomycota (*Olpidium brassicae*). In contrast to bacteria/archaea, fungi were also influenced by N-fertilization intensity. Int enriched two species (with highest affiliation to *Ascomycota* sp. and *Minimedusa polyspora*) while in Ext six species with highest similarity to *Pseudogymnoascus appendiculatus, Talaromyces veerkampii, Hypocreaceae* sp., *Mortierella alpina, Olpidium brassicae* (same ASV like in MP), and *Filobasidium oeirense* exhibited significantly higher relative abundances (Table S12, Supporting Information).

**Table 7.** Fungal species (ASVs) with differential relative abundance in the rhizosphere (RH) of winter wheat (cv. Lemmy, EC 59) grown in mouldboard plough (MP) or cultivator tillage (CT) soils (FDR < 0.05). Only discriminative species with mean > 0.5% are displayed. Significantly enriched taxa are marked in bold. Mean ± standard deviation is shown (*n* = 8), unident. – unidentified, inc. sed. – incertae sedis.

Phylum	Genus	ASV	SH-No.	MP	CT
Ascomycota	*Ascomycota_unident*.	*Ascomycota sp*	SH1555467.08FU	**2.0 ± 1.0**	0.3 ± 0.1
Ascomycota	*Chrysosporium*	*Chrysosporium lobatum*	SH1552992.08FU	**1.3 ± 0.9**	0.5 ± 0.3
Ascomycota	*Pseudogymnoascus*	*Pseudogymnoascus pannorum*	SH2267912.08FU	**1.4 ± 0.7**	0.2 ± 0.1
Ascomycota	*Acremonium*	*Acremonium persicinum*	SH1513361.08FU	**0.6 ± 0.2**	0.1 ± 0
Basidiomycota	*Strophariaceae_unident*.	*Strophariaceae* sp.	SH1639424.08FU	**1.2 ± 2.1**	0.1 ± 0.1
Basidiomycota	*Sporidiobolaceae_unident*	*Sporidiobolaceae* sp.	SH1575137.08FU	**1.3 ± 0.7**	0.4 ± 0.3
Mortierellomycota	*Mortierella*	*Mortierella fimbricystis*	SH1608147.08FU	**0.9 ± 0.6**	0.1 ± 0
Mortierellomycota	*Mortierella*	*Mortierella antarctica*	SH1650287.08FU	**0.5 ± 0.4**	0.1 ± 0
Olpidiomycota	*Olpidium*	*Olpidium brassicae*	SH1519091.08FU	**1.0 ± 1.5**	0 ± 0
Ascomycota	*Leptosphaeriaceae_unident*.	*Leptosphaeriaceae* sp.	SH1635388.08FU	0.2 ± 0.2	**1.2 ± 0.6**
Ascomycota	*Penicillium*	*Penicillium aethiopicum*	SH2190001.08FU	0.6 ± 0.3	**1.7 ± 0.5**
Ascomycota	*Articulospora*	*Articulospora* sp.	SH1648789.08FU	0.7 ± 0.6	**2.0 ± 1.4**
Ascomycota	*Chaetomium*	*Chaetomium* sp.	SH1615796.08FU	1.6 ± 2.3	**3.0 ± 0.9**
Ascomycota	*Trichoderma*	*Trichoderma petersenii*	SH2303584.08FU	0.4 ± 0.2	**2.0 ± 1.8**
Ascomycota	*Trichoderma*	*Trichoderma piluliferum*	SH2305819.08FU	0.5 ± 0.4	**1.8 ± 0.8**
Ascomycota	*Fusarium*	*Fusarium* sp.	SH1610194.08FU	0.2 ± 0.2	**0.6 ± 0.4**
Ascomycota	*Stachybotrys*	*Stachybotrys chartarum*	SH1557965.08FU	2.2 ± 1.9	**5.1 ± 2.5**
Basidiomycota	*Tausonia*	*Tausonia pullulans*	SH1650607.08FU	0.3 ± 0.2	**0.7 ± 0.4**
Mucoromycota	*Rhizopus*	*Rhizopus arrhizus*	SH1510152.08FU	1.5 ± 0.8	**4.9 ± 2.1**

### Functional profiling of the RH microbiome

**Figure 3.**
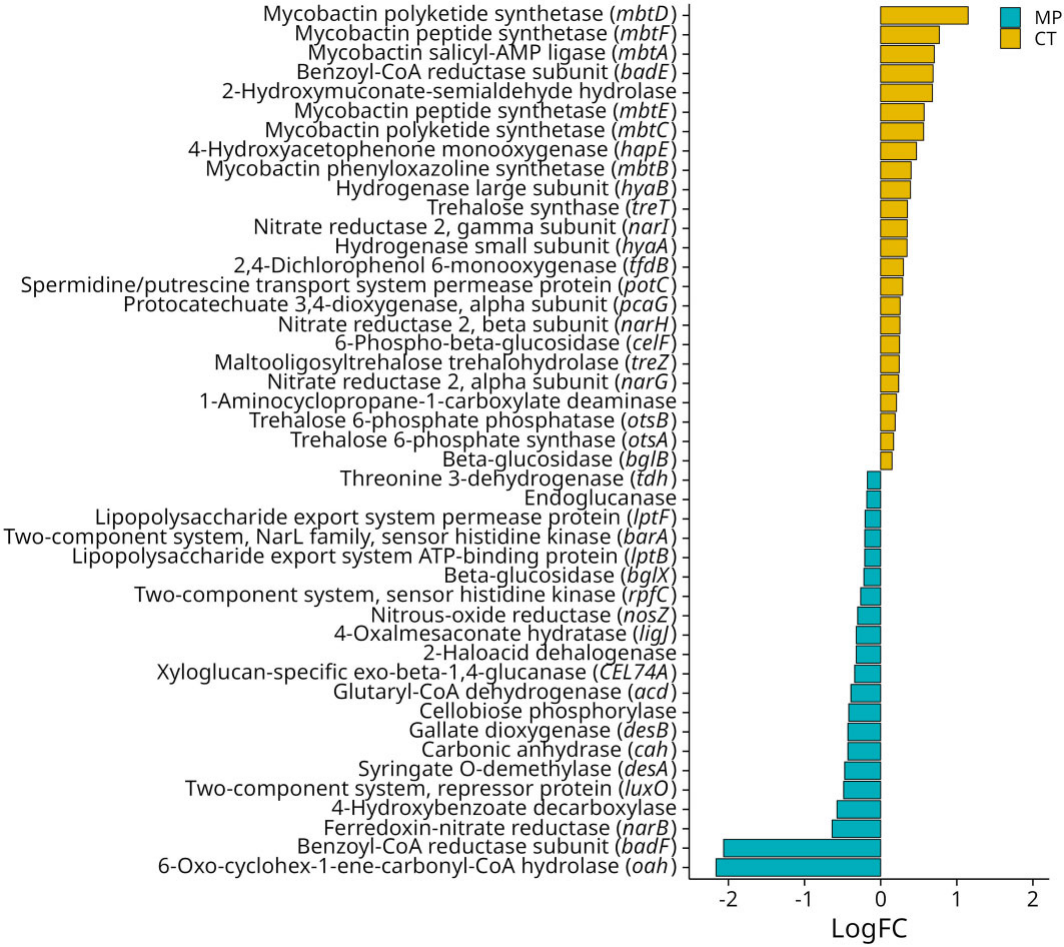
Differentially abundant plant-beneficial functions (customized database) in the rhizosphere (RH) of winter wheat (cv. Lemmy, EC 59) grown in mouldboard plough (MP) vs. plants grown in cultivator tillage (CT) soils based on edgeR. Significantly enriched functions are marked in orange (CT) or blue (MP). Mean  ± standard deviation is shown (*n* = 8).

Tillage was the main driver for bacterial plant-beneficial functions, followed by the combination of tillage and N-fertilization intensity (Fig. 2C; Table 4). A total of 350 genes were detected based on the customized database of plant-beneficial functions. From these, 45 were differentially abundant with 24 genes enriched in CT and 21 enriched in MP treatments. Under CT, genes coding for ACC deaminase, siderophore production, spermidine, and trehalose synthase were enriched in RH (Fig. 3). In contrast, genes for biofilm production and quorum sensing exhibited significantly higher relative abundances in the RH of MP plants (Fig. 3). In RH of both tillage practices, different genes encoding for N-metabolism, aromatic compound degradation and secondary metabolite production were differentially abundant.

**Figure 4.**
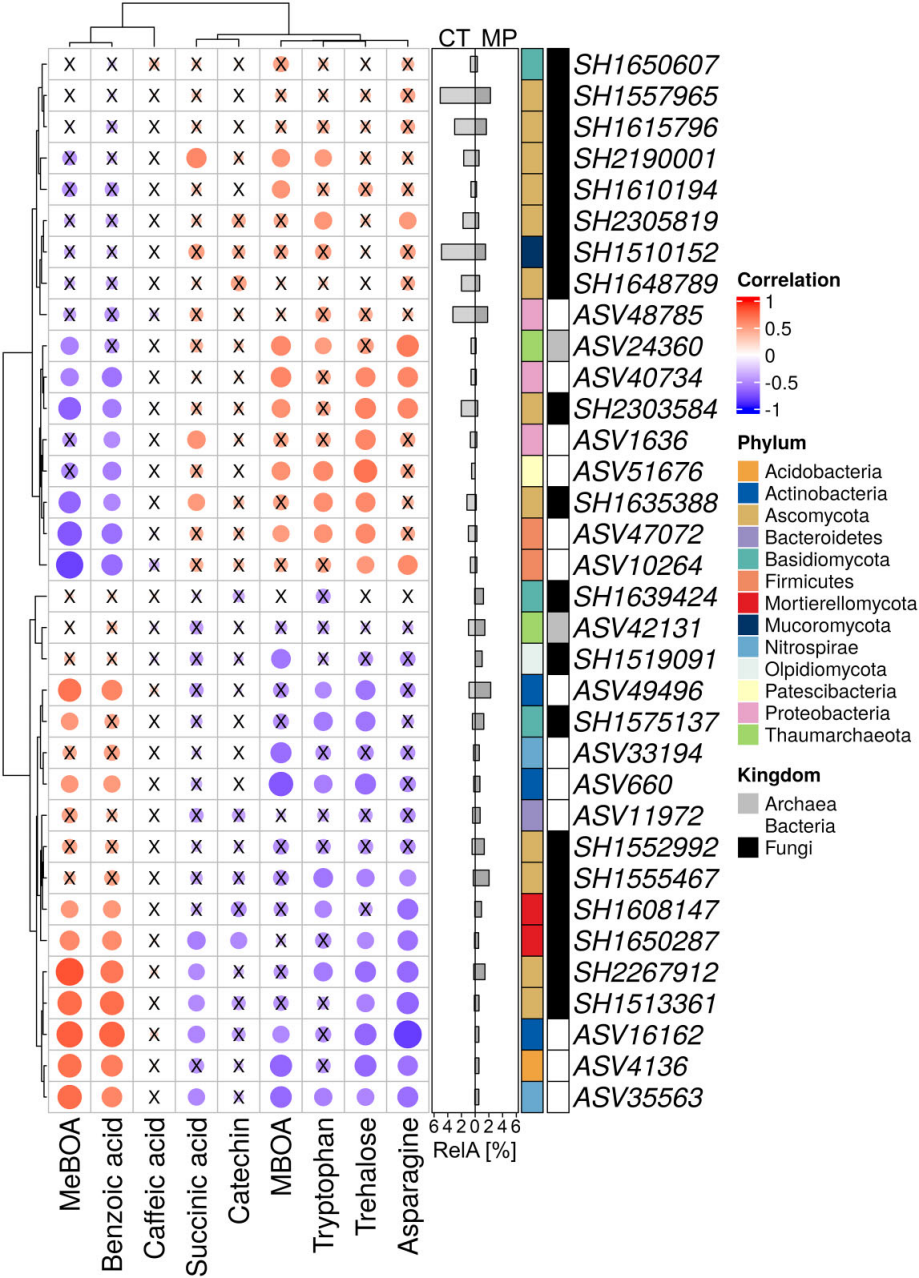
Correlation (Kendall) matrix of microbial responders [relative abundance (RelA) > 0.5%] and metabolites in rhizosphere (RH) soil solution of winter wheat (cv. Lemmy, EC 59) apical roots. Only the metabolites affected by the different long-term agricultural practices are shown (Table 5). Organisms and metabolites are sorted according to correlation-based hierarchal cluster analysis. High positive correlations are represented by dark red, negative ones by dark blue circles, whereas the circle diameter is indicative for the strength of the correlation. X, not significantly correlated (tau < 0.5 or > −0.5 with a *P* > .01). The effect of tillage practice on the mean RelA of microbial responders is shown in a bar chart (cultivator tillage [CT] = light gray and mouldboard plough [MP] = dark gray).

For linking functions with taxonomy, sequences of the differentially abundant functions were extracted and taxonomically classified. In order to explore whether they contributed to the positive plant–microorganism interactions observed under CT (Tables 1 and 3, Fig. 4), the focus was put on 12 genes associated with functions exclusively increased in CT treatments (Figure S8, Supporting Information). These were genes encoding ACC deaminase and *mbtA, mbtB, mbtC, mbtD, mbtE*, and *mbtF*, encoding the siderophore mycobactin, *potC* involved in spermidine production, as well as *otsA, otsB, treT*, and *treZ* involved in trehalose production. Between 50% and 70% of the extracted sequences were classified on genus level. Among the ten most detected genera associated with these genes, the majority belonged to Actinobacteria/Actinomycetota, such as *Mycolicibacterium, Nocardia, Nocardioides, Rubrobacter*, and *Streptomyces* (Figure S8, Supporting Information). Furthermore, these genes were associated with the genera *Devosia, Mesorhizobium*, and *Microbacterium*, which were also detected as positive responders in CT based on 16S amplicon sequencing.

### Correlation between microbial responders and RH metabolites

The correlation analysis between microbial responder taxa and metabolites in the RH soil solution showed two main clusters (Fig. 4). Cluster 1 contained fungal, archaeal, and bacterial taxa that were positively correlated with succinic acid, catechin, MBOA, tryptophan, trehalose, and asparagine. Notably, fungal ASVs of cluster 1 mainly belonged to Ascomycota and were affiliated among others to *Trichoderma* spp. and *Penicillium* spp. (Fig. 4; Table S14, Supporting Information). Bacterial ASVs of cluster 1 were affiliated to Proteobacteria, Patescibacteria, and Firmicutes (e.g. *Bacillus*). In contrast, cluster 2 contained bacterial and fungal taxa, which were positively correlated with MeBOA and benzoic acid and negatively with the other compounds. The bacterial ASVs were affiliated to e.g. Actinobacteria, Nitrospirae, and Acidobacteria (Fig. 4; Table S13, Supporting Information). For fungal ASVs, this was true for representatives of the Ascomycota and Mortierellomycota.

## Discussion

### Reduced N-fertilization intensity supported wheat root development

The root development is strongly influenced by nutrient availability and fertilization (Pierret et al. 2007). Bilalis et al. (2015) showed that reduced nutrient availability stimulates root properties required for nutrient acquisition. In the present study, winter wheat under Ext exhibited a larger root system compared to Int, regardless of the tillage practice, likely supporting the uptake of water and nutrients even under reduced availability.

The reductive assimilation of nitrate as the predominant N source in mineral fertilized soils requires the successive activities of nitrate reductase 1 (*TaNR1*) and nitrite reductase (*TaNIR*) for conversion of nitrate to nitrite and finally to ammonium (Costa-Broseta et al. 2021). The higher amount of available N consistently induced higher expression of these genes, including the nitrate transporter gene (*TaNPF7.1*, NRT1-PTR-Family), in the Int compared to Ext treatments (Figure S4, Supporting Information). Aside from K, which was below the deficiency level across all treatments, there were no specific nutrient deficiencies. Consequently, winter wheat plants acquired sufficient nutrient amounts, leading to comparable shoot biomass across treatments regardless of N-fertilization intensity (Table 1). This highlights that wheat compensated the reduced N availability (Ext) by extending the root architecture and physiological properties. As wheat under Ext and reduced tillage (CT) produced the same yield like plants under Int and MP, current N-fertilization practices for wheat still appear too high, with potential negative consequences for the environment, due to leakage.

### Agricultural practice altered RH metabolites and plant–microorganism interactions

Root exudates and related RH metabolites are key drivers of interactions at the plant–microorganism–soil interface (Hu et al. 2018, Kudjordjie et al. 2019, Oburger et al. 2022) influenced by various factors (Neumann and Ludewig 2023). The root window setup combined with microsampling of RH soil solutions (Neumann 2006) allowed to track spatial variations along different root zones and relate them to the RH microbiome and plant performance. In our study, tillage practice, but not N-fertilization intensity, predominantly influenced RH metabolite patterns, especially in the young apical root zones (Fig. 1B).

Tryptophan was significantly increased in the RH of plants grown under CT (Table 5). Many RH microorganisms use tryptophan as a precursor for indole acetic acid (IAA) production (Spaepen and Vanderleyden 2011, Vurukonda et al. 2016) and thereby stimulate the growth of fine lateral roots, as observed also in this study (Table 1). Elevated tryptophan concentrations were also positively correlated with an enrichment of *Bacillus* in the RH (Fig. 4, Table 6), containing strains described to produce IAA (Özdal et al. 2016). Interestingly, microbial genes involved in IAA production were not among the differentially abundant genes (Fig. 3). However, it has been reported that microorganisms can also improve lateral root formation independent of IAA production (Yu et al. 2021) by modulating the hormonal balances of the host plant via changes in plant hormonal metabolism (Moradtalab et al. 2020). In this context, also the increased abundance of ACC deaminase genes in RH of CT plants could be relevant for the stimulation of fine root development (Table 1) by lowering the plant ethylene level acting as an antagonist (Glick et al. 2007).

Other RH metabolites, such as succinic acid, trehalose, and asparagine, were higher in the CT treatments and correlated with the increased relative abundance of potentially beneficial microorganisms (*Bacillus* and *Trichoderma*; Fig. 4). Succinic acid acts as a chemoattractant and carbon source for various beneficial microorganisms and is relevant for functions in biocontrol and plant defense responses (Sampedro et al. 2015). Trehalose represents a signaling molecule regulating bacterial and fungal growth, development, and virulence (Sharma et al. 2020), but it is also produced by microorganisms in high quantities, promoting adaptive responses to abiotic stress in plants (Vurukonda et al. 2016, Kosar et al. 2019). Moreover, trehalose can induce resistance to powdery mildew in wheat (Reignault et al. 2001), which is in line with the here observed reduced symptoms of powdery mildew in the CT treatments. The increased abundance of trehalose synthase genes in the RH of CT plants (Fig. 3) suggests a microbial origin for the elevated trehalose levels.

Apart from some phenolic acids and flavonoids, the predominant secondary metabolites detected in the RH soil solution were different BXs, derived from the young apical root zones particularly of CT plants. These compounds are produced by most cereal crops at an early growth stage and in response to stress events as defense substances with allelopathic, insecticidal (antifeeding), and antimicrobial effects (Hu et al. 2018, Kudjordjie et al. 2019, Schandry and Becker 2020). Additionally, they can stimulate growth of beneficial soil microorganisms, decrease plant growth of competitors, increase JA signaling and plant defenses, and suppress herbivore performance in the next plant generation (Hu et al. 2018, Oburger et al. 2022). In soils, these highly bioactive substances are rapidly converted into more stable derivates, such as APO, MBOA, MeBOA, and AMPO, through both microbial and nonmicrobial processes (Schütz et al. 2019, Oburger et al. 2022). In the CT treatments, MBOA was predominant, while in the MP treatments, other BXs (MeBOA) dominated, which could indicate differences in microbial degradation between CT and MP tillage practice. The accumulation of MBOA was positively correlated with potentially antagonistic microorganisms (*Bacillus* and *Trichoderma*) (Tables 6 and 7; Fig. 4). This, together with the fact that MBOA has toxic effects on various fungal pathogens (Cotton et al. 2019, Kudjordjie et al. 2019), such as *Fusarium* species (Glenn et al. 2001), may explain the reduced relative abundance of putative plant pathogenic fungal genera in the CT-Ext treatment (*Zymoseptoria* and *Gibellulopsis*; Figure S5B, Supporting Information).

### Agricultural practices altered the RH microbiome of winter wheat

The RH microbiome strongly depends on the soil microbiome, which is influenced by different factors, such as agricultural practice (Bulgarelli et al. 2013). In our study, tillage practice was the main factor affecting the microbiome in the RA soil and RH (Table 4), indicating that the complete plant–microorganism–soil system was affected. These observations are in line with results from previous studies investigating wheat soils (Sommermann et al. 2018, Babin et al. 2019, Romano et al. 2023), which highlights the stable legacy of tillage practice likely caused by differences in physical soil properties (Schlüter et al. 2018) as well as differences in chemical composition (e.g. C and N stocks; Table S15, Supporting Information). N-fertilization intensity shaped the communities only to a minor extent with a stronger influence on fungi. This confirmed previous studies (Sommermann et al. 2018, Bziuk et al. 2021) and could be related to the availability of N that influences the activity and growth efficiency, especially of saprotrophic fungi (Di Lonardo et al. 2020). Clearly, not only plants can influence the microbial community via root exudation, but microorganisms can also alter the exudate composition (Korenblum et al. 2020). Our results further support such interdependence of root exudates and the microbiome (Fig. 4).

The effects of tillage practice on the composition of root exudates are likely related to specific patterns of substrate utilization by the microbiome. Bacterial and archaeal species in the RH of MP plants belonged mainly to Actinobacteria and Acidobacteria (Table 6; Table S10, Supporting Information), which are typically regarded oligotrophs (Fierer 2017). This might indicate that the selective effect of the plant is lower in MP than in CT. The acidobacterial genus RB41 has been frequently isolated from soil and was reported as a tillage responder. In accordance with our results (Table S10, Supporting Information), Kudjordjie et al. (2019) found a negative correlation between many acidobacterial species and BX compounds in maize roots. BXs have a selective impact on root and RH microbiota across different field locations (Niemeyer 2009, Kudjordjie et al. 2019, Cadot et al. 2021). Taxa with higher relative abundance in the RH of CT plants compared to MP plants belonged to typical RH genera such as *Bacillus, Sphingomonas, Devosia*, and *Pseudoxanthomonas* (Table 6; Table S10, Supporting Information), which are known to harbor members with plant-beneficial properties (Chowdhury et al. 2019). Additionally, many sequences with affiliation to *Saccharimonadaceae* (Patescibacteria) were enriched in the RH of CT plants. A symbiotic lifestyle and cometabolism interdependencies were proposed for Patescibacteria (Lemos et al. 2019), which might favor these taxa in microbial hotspots such as the RH. In summary, this suggests that the driving effect of the plant on the soil bacterial/archaeal community was stronger in soils under CT than MP tillage.

In the RH of CT more fungal genera with known plant-beneficial members, such as *Trichoderma*, were present, whereas potential plant pathogens, like *Olpidium*, were more abundant in the RH of MP plants. The genus *Olpidium* harbors typical pathogens for Brassicaceae (Lay et al. 2018) like rapeseed, which was the previous crop at the sampling site. Furthermore, *Chrysosporium* species were enriched in MP and are known in the context of gibberellin production, which can enhance plant growth (Hamayun et al. 2009), but is detrimental for plants in high doses (Cen et al. 2020). Among the fungal responders in CT were many potential fungal antagonists such as *Chaetomium, Penicillium, Trichoderma*, and nonpathogenic *Fusarium*, which were often found in disease-suppressive soils (reviewed by van Bruggen and Semenov 2000, Mazzola 2002). *Trichoderma* species are well known as plant-growth promoters, mycoparasite and inducers of plant systemic resistance against pathogens (Harman et al. 2004, López-Bucio et al. 2015, Hafiz et al. 2023) and were previously identified in a growth chamber experiment with soils from the identical sampling site as responders to CT (Babin et al. 2021). The class Sordariomycetes was increased in the RH of CT plants with high BXs concentration (Fig. 4), which confirms previous studies (Kudjordjie et al. 2019).

The effect of tillage on microbial communities was not only observed on a taxonomic but also functional level. ACC deaminase activity, siderophore, trehalose, and spermidine production were enriched in the RH of CT plants (Fig. 3). ACC deaminase lowers the level of the plant stress hormone ethylene and is, therefore, considered as beneficial (Dubois et al. 2018). Genes encoding ACC deaminase were present in several genera including *Microbacterium*, which was enriched in CT samples and is well-known to harbor plant-beneficial members (Vílchez et al. 2018, Freitas et al. 2019). Moreover, spermidine is a compound with several important functions, e.g. biofilm production, overall bacterial fitness, plant growth promotion, and stress protection (Alavi et al. 2013, Xie et al. 2014, Chen et al. 2018). Among the classified genera, that encode spermidine production, was the responder genus *Mesorhizobium* from the RH of CT. In rhizobia, spermidine plays a role in symbiotic interactions and root nodule formation (Becerra-Rivera and Dunn 2019). Siderophores are iron-chelating compounds that are involved in pathogen suppression by iron competition (Gu et al. 2020) or by inducing systemic resistance (Zamioudis et al. 2015, Verbon et al. 2019). Several genera were associated with siderophore production, but no responder genus was included (Figure S8, Supporting Information). Thus, the increased siderophore production in CT plants seems to be a concerted effect of several beneficial taxa. Comparing amplicon and metagenomics data, responders encoding trehalose synthase genes were found (*Microbacterium* and *Devosia*; Figure S8 and Table S10, Supporting Information). Trehalose produced by *Microbacterium* sp. 3J1 was previously described to be involved in the protection of pepper plants against drought stress (Vílchez et al. 2018). Taken together, these responders could have contributed to the observed improved plant health in CT plants.

### Reduced tillage practice and N-fertilization intensity enhanced stress tolerance in winter wheat

Analysis of stress-related metabolites and genes in wheat leaves highlighted that tillage practice had a pronounced effect on metabolic stress adaptation. In contrast to MP plants, lower concentrations of stress hormones (JA and SA) and stress indicators (T-AO, proline, and APX; Table 3) were revealed for CT plants. Moreover, increased abundance of the ACC deaminase gene derived from bacteria in the RH of CT plants (Fig. 3) could have also contributed to the reduced stress responses in CT plants (Glick et al. 2007).

The elevated stress level of MP plants was underlined by the differentially enriched expression of genes involved in stress responses (Fig. 1A) like the well-characterized pathogenesis-related gene *PR1* (β-1,3-glucanase) and Chitinase (*TaCHI*). These genes encode for proteins involved in hydrolysis of glucan and chitin, which are present in fungal cell walls (van Loon et al. 2006) and have been shown to be upregulated in *Puccinia triticina* infected wheat leaves (Casassola et al. 2015) and in *Pyricularia oryzae* infected rice leaves (Cruz et al. 2015). In our study, an enhanced expression of other defense-related genes like lipoxygenase (*TaLOX*), defensin (*Tad1*), and allene oxide synthase (*TaAOS*), which are highly inducible by biotic stress factors (Manners et al. 1998), was observed. The cross-talk among SA, JA, and ET in the regulation of plant stress responses has been extensively studied in model plants like *Arabidopsis* (Pieterse et al. 2009, Verhage et al. 2010).

The increased expression of *TaSOD, TaCAT*, and *TaPER* genes associated with leaf accumulation of T-AO and increased APX activity in the MP plants indicates an activation of defense mechanisms to detoxify free radicals and to alleviate the oxidative damage associated with the overproduction of reactive oxygen species (Miller et al. 2010). Our findings from wheat leaf gene expression suggest that compared to CT, plants grown in MP soil showed enhanced activity of defense signaling pathways, potentially triggered by the presence of a leaf pathogen (i.e. *B. graminis*). This observation was supported by an elevated concentration of the stress hormones JA and SA in the shoots (Table 3). The described scenario indicates a reduced influence of stress factors on wheat plants grown in CT soil, which was most likely supported by modifications of the interactions with the RH microbiome e.g. via root exudates.

## Conclusion

In our study, we used a long-term field study site and in-field root windows to obtain a holistic view on the impact of agricultural practice (tillage, N-fertilization intensity) on the complex interaction between plant roots and the surrounding microbial community in soil. Tillage practice strongly impacted on the multipartite interaction network with consequences for plant health and stress resilience. CT combined with reduced N-fertilization intensity resulted in a positive plant–microorganism feedback in the RH contributing to an improved plant performance compared to conventional management. However, care should be taken as these observations cannot be generalized as indicated by long-term (2012–2016) higher yields in the conventional (9.6 t ha^−1^ MP-Int) compared to conservation practice (8.4 t ha^−1^ CT-Ext). We, therefore, suggest that the observed compensatory effects of conservation practices are more likely to play a role under unfavorable conditions such as pathogen pressure or drought, which are predicted to occur more frequently in a changing climate. Altogether, this study contributes to a holistic understanding of soil–plant–microorganism interactions in agricultural settings.

## Supplementary Material

fiae003_Supplemental_Files

## Data Availability

Unassembled raw ITS sequences were submitted to European Nucleotide Archive (ENA; BioProject accession number PRJEB67553) and unassembled raw 16S rRNA gene sequences and shotgun metgenome sequences were submitted to NCBI Sequence Read Archive (SRA; Bioproject accession number PRJNA1006757 [shotgun metagenome] and PRJNA1037063 [16S rRNA gene amplicon sequencing]).
