# Supplementary material for: Long-term conservation tillage with reduced nitrogen fertilization intensity can improve winter wheat health via positive plant–microorganism feedback in the rhizosphere
Source: FEMS Microbiol Ecol. 2024 Jan 15;100(2):fiae003. doi: 10.1093/femsec/fiae003 (PMC10847717; doi:10.1093/femsec/fiae003)
Supplement: fiae003_Supplemental_Files [file fiae003_supplemental_files.zip › Supplement_data MS2_Final_LS_db_clean.pdf]

Supplement

Long-term conservation tillage with reduced nitrogen fertilization intensity can improve winter wheat health via positive plant-microorganism feedback in the rhizosphere

Jan Helge Behr[1#], Theresa Kuhl-Nagel[1#], Loreen Sommermann[2#], Narges Moradtalab[3], Soumitra Paul Chowdhury[4], Michael Schloter[4], Saskia Windisch[3], Ingo Schellenberg[2], Lorrie Maccario[5], Sören J. Sörensen[5], Michael Rothballer[4], Joerg Geistlinger[2], Kornelia Smalla[6], Uwe Ludewig[3], Günter Neumann[3], Rita Grosch[1#], Doreen Babin[6#*]

Supplement Text 1: Detailed field work calendar for cultivation of winter wheat after rapeseed in the long-term field trial Bernburg, growing season 2018/2019. MP- mouldboard plough tillage, CT- cultivator tillage, Ext- extensive N-fertilization intensity without fungicides and growth regulators, Int- intensive N-fertilization intensity with pesticides and growth regulators.

09 July 2018	mulcher
08 August 2018	disc harrow
16 August 2018	basic fertilizer
09 October 2018	MP tilled
09 October 2018	CT: 2x heavy cultivator
09 October 2018	light cultivator
10 October 2018	sowing with Amazone 300 seeds m^{-2}
20 October 2018	MP: emergence of plants
21 October 2018	CT: emergence of plants
26 October 2018	milled in
16 November 2018	plants counted
21 February 2019	soil sampling for Nmin
26 February 2019	herbicide treatment 70 g ha^{-1} Biathlon HD + 1.0 L ha^{-1} Dash + 20 g ha^{-1} Dirigent
07 March 2019	1st Nitrogen fertilization: Int 60 kg N ha^{-1} Domogran 45, Ext 30 kg N ha^{-1} Domogran 45
08 April 2019	Int: growth regulators 1.0 L ha^{-1} CCC_{720}
**09 April 2019**	**Root window installation**
15 April 2019	2nd Nitrogen fertilization: Int 70 kg N ha^{-1} HS, Ext 30 kg N ha^{-1} HS
08 May 2019	Int: fungicide treatment 1.0 L ha^{-1} Priaxor + 1.0 L ha^{-1} Osiris
**21 May 2019**	**Root window sampling**
23 May 2019	Int: 3rd Nitrogen fertilization 40 kg N ha^{-1} KAS
04 June 2019	Int: 0.5 L ha^{-1} Taspa +0.5 L ha^{-1} Folicur + 0.075 L ha^{-1} Karate Zeon
23 July 2019	harvest
26 July 2019	soil sampling for Nmin

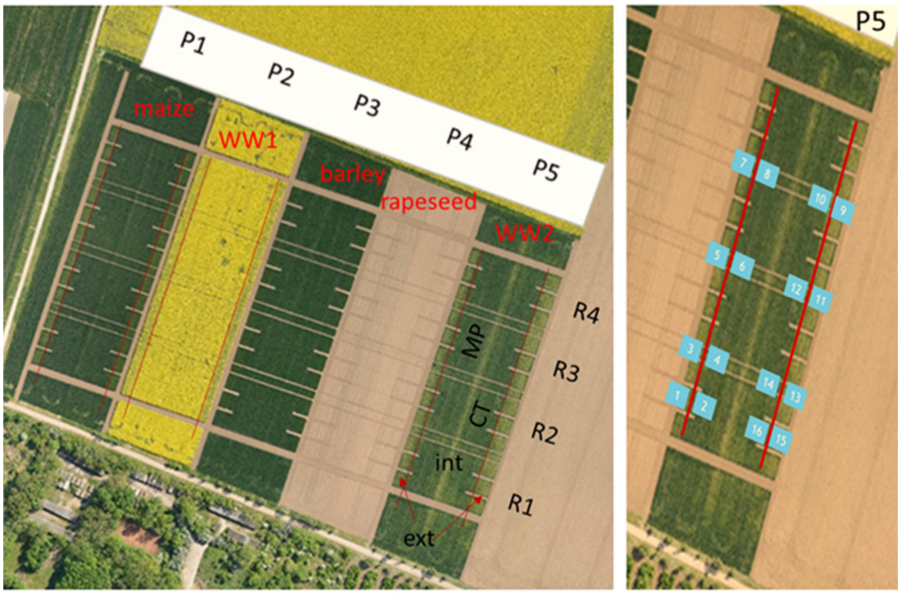

Figure S1. Aerial view on the long-term field trial Bernburg (left) and installation positions of root windows in winter wheat 2 (cv. Lemmy) grown under cultivator tillage (CT) or mouldboard plough (MP) with either intensive (Int) or extensive N-fertilization intensity (Ext), (Source: Anhalt University of Applied Sciences).

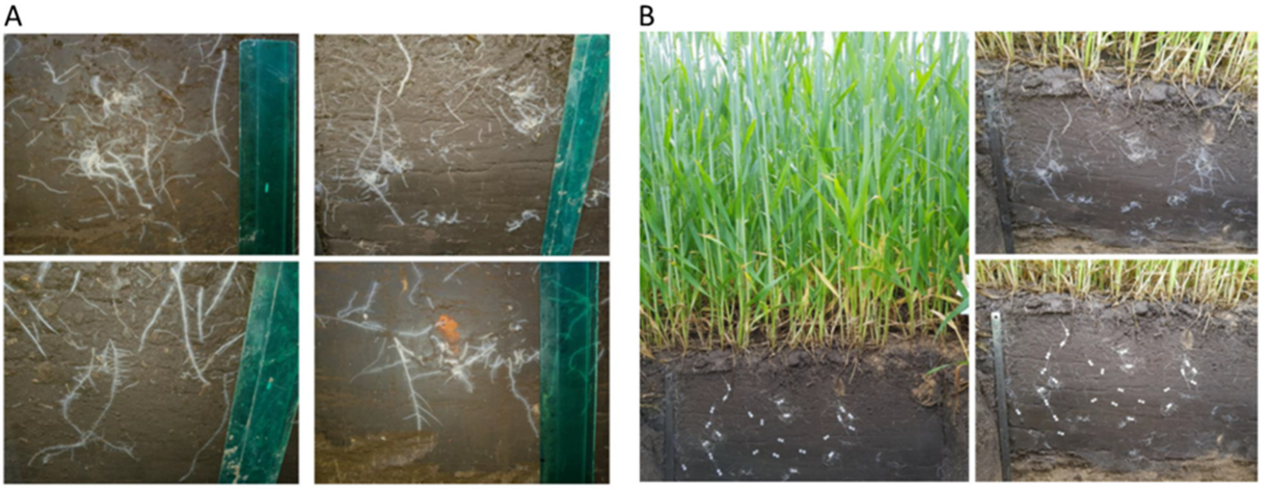

Figure S2. A) Root development of winter wheat (cv. Lemmy) along the root window 42 days after installation (EC 59). B) Sorption filter sampling.

Table S1. List of wheat (*Triticum aestivum*) genes selected for qRT-PCR analysis, their corresponding primer pairs, amplification efficiency (AE, calculated from amplification curve) and references.

**Gene**	**Forward P (5′ to 3′)**<br>**Reverse P (5′ to 3′)**	**AE (%)**	**Reference**
TaUBIQ (Ubiquitin)	CCTTCACTTGGTTCTCCGTCT<br>AACGACCAGGACGACAGACACA	91.64	Cruz et al. 2015
TaEF1α (Elongation factor 1α)	ATGATTCCCACCAAGCCCAT<br>ACACCAACAGCCACAGTTTGC	96.20	Cruz et al. 2015
TaPR1 (β-1,3-glucanase HQ848391)	CAATAACCTCGGCGTCTTCATCAC<br>TTATTTACTCGCTCGGTCCCTCTG	97.87	Casassola et al. 2015
TaLOX (Lipoxygenase)	CGACCCGCAGCTGTTGA<br>CCCTTGTGATCGGAGGTGTT	94.75	Cruz et al. 2015
Tad1 (AB089942 Defensin)	GCAGAGCCACAAGTTCAAGG<br>GTCCGCTTGCAGTAGCACTT	93.49	This study
TaAOS (Allene Oxide Synthase)	ACCGTGTTCAACAGCTACGG<br>AGCGCCTCTATCGTCACCTT	97.87	Wang et al. 2017
TaCHI (Chitinase)	CTGGACCTGGACTTCGACAC<br>CGCTTGAGCACGGGTTAAT	94.34	Cruz et al. 2015
TaSOD (JQ613154.1 Superoxide Dismutase)	CATTGTCGATAGCCAGATTCCTTT<br>AGTCTTCCACCAGCATTTCCAGTA	98.96	Zhang et al. 2016
TaCAT (GU984379.1 Catalase)	TTTGATGGGAGTCTTGTGCTTGTG<br>ACGGTGAGGGAGTTGTCGTTGTT	94.74	Zhang et al. 2016
TaPOX (Peroxidase X53675.1)	CAGCCCTGTAGCCAACATAAA<br>GCACTTCCACGACTGCTTTG	96.28	Zhang et al., 2016
TaRubiscoSSU (ribulose bisphosphate carboxylase/oxygenase small subunit on Chr5)	GGTGGAGGAGGTCAAGAAGGAG<br>MGTCGTGAGTGAGCTGTTTAGGC	92.11	Buchner and Hawkesford, 2014
TaPIE1 (EF583940) (ERF transcription factor)	GGAGCCACCAGTCCGTATGA<br>CACCCGGCAGAGGTATTCAA	92.03	Zhu et al. 2014
*TaGSTU*4 (Glutathione-S-transferase 4)	TTCAAGCATCCAACCTCTCC<br>GCTGTCACATCCATCCAAAA	97.88	This study
TaGSTZ (Glutathione-S-transferase)	CCAAGCCCATTTGTTACCAG<br>GTGGATGAGCACGGGTATCT	90.37	This study
TaPIMP2- (MYB Transcription Factor)	GCATTGTACGGCCAGTTCG<br>CGAGGAGGCTCTGTTCTTGG	93.72	Wei et al. 2017b
TaMYB80 (MYB Transcription Factor 80)	CAGATGCTCCTCCCTTGG<br>GTGATCCTGGTGTAGTTGC	91.15	Zhao et al. 2017
TaODORANT1 (MYB Transcription Factor)	CCGAAGCCCATGTACCTCC<br>CGGATCTATGATCGGTCTATGTG	103.99	Wei et al, 2017a
TaWRKY49 (WRKY transcription factor)	CTTCCCTGCCGCATTCT<br>ACGCTCTCGCCCTAGTG	95.4	Wang et al. 2017
TaWRKY62 (WRKY transcription factor)	TCGTTGACCACCACCAG<br>AGCCGTCCCCAAATCCA	91.22	Wang et al. 2017
TaNR1 (Nitrate Reductase)	GGCCAATTCYTTCATCTCCTTCTG<br>TACRTSCACAGATTGATGCGTCSA	92.06	Buchner and Hawkesford 2014
TaNIR (Nitrite reductase)	ACGAGGAGTAGGCCGGCTASGAG<br>ATCAGCCGCAGCCCATCTCTRC	90.47	Buchner and Hawkesford 2014

TaNPF7.1 (nitrate transporter/Peptide transporter family)	CTACAAGACCTGCGCCATCTTC<br>GATGAGGTATAGCCGCGAGGAG	91.60	Buchner and Hawkesford 2014
TaGDH2 (Glutamate dehydrogenase 2)	AGGATGGGAGCATTCACCTTGG<br>GGATATAAGAACTKTCATCCACCACG	94.56	Buchner and Hawkesford 2014
TaVIT2 (Iron transporter)	CTCCCCCTACATGTTCGT<br>CCCTTGACGTAGCCGAA	95.07	Connorton et al. 2017
TaMPK3 (Mitogen activated Protein Kinase 3)	GGAGATCAAGCTCCTCAGGC<br>ACTGGCAGTGTTCTTCCGAG	90.99	Goyal et al. 2018
TaMPK4 (Mitogen activated Protein Kinase)	TCGAGCCTGGGATTTCTTCG<br>GTCAACAGTGATGCGTCTGC	91.56	Goyal et al. 2018
TaMPK6 (Mitogen activated Protein Kinase)	CAGCTTATCTCCGAGGAAAACG<br>TTGTGCCGCACTAGTTGGA	94.18	Goyal et al. 2018
TaMKK6 (Mitogen activated Protein Kinase Kinase)	CAGCTTATCTCCGAGGAAAACG<br>TTGTGCCGCACTAGTTGGA	92.16	Goyal et al. 2018
TaMKK10-1/3a (Mitogen activated Protein Kinase Kinase)	CTCAAGGTCCAGCACTACGG<br>CCACCAGCTCGAGGAGTAGA	91.78	Goyal et al. 2018
TaMKK10-1/ 3b (Mitogen activated Protein Kinase Kinase)	CCAGGGCCCTATGATCCGTA<br>TTGCCGAAGAAGATAGCGCA	100.07	Goyal et al. 2018

Buchner, P. and Hawkesford, M.J., 2014. Complex phylogeny and gene expression patterns of members of the NITRATE TRANSPORTER 1/PEPTIDE TRANSPORTER family (NPF) in wheat. *Journal of experimental botany*, *65*(19), pp.5697-5710.

Casassola, A., Brammer, S.P., Chaves, M.S., Martinelli, J.A., Stefanato, F. and Boyd, L.A., 2015. Changes in gene expression profiles as they relate to the adult plant leaf rust resistance in the wheat cv. Toropi. *Physiological and Molecular Plant Pathology*, *89*, pp.49-54.

Connorton, J.M., Jones, E.R., Rodríguez-Ramiro, I., Fairweather-Tait, S., Uauy, C. and Balk, J., 2017. Wheat vacuolar iron transporter TaVIT2 transports Fe and Mn and is effective for biofortification. *Plant Physiology*, *174*(4), pp.2434-2444.

Cruz, M.F., Debona, D., Rios, J.A., Barros, E.G. and Rodrigues, F.A., 2015. Potentiation of defense-related gene expression by silicon increases wheat resistance to leaf blast. *Tropical Plant Pathology*, *40*(6), pp.394-400.

Goyal, R.K., Tulpan, D., Chomistek, N., Fundora, D.G.P., West, C., Ellis, B.E., Frick, M., Laroche, A. and Foroud, N.A., 2018. Analysis of MAPK and MAPKK gene families in wheat and related Triticeae species. *BMC genomics*, *19*(1), p.178.

Wang, J., Tao, F., Tian, W., Guo, Z., Chen, X., Xu, X., Shang, H. and Hu, X., 2017. The wheat WRKY transcription factors TaWRKY49 and TaWRKY62 confer differential

high-temperature seedling-plant resistance to *Puccinia striiformis* f. sp. *tritici*. *PloS one*, *12*(7), p.e0181963.

Wei, Q., Luo, Q., Wang, R., Zhang, F., He, Y., Zhang, Y., Qiu, D., Li, K., Chang, J., Yang, G. and He, G., 2017a. A wheat R2R3-type MYB transcription factor TaODORANT1 positively regulates drought and salt stress responses in transgenic tobacco plants. *Frontiers in plant science*, *8*, p.1374.

Wei, X., Shan, T., Hong, Y., Xu, H., Liu, X. and Zhang, Z., 2017b. TaPIMP2, a pathogen-induced MYB protein in wheat, contributes to host resistance to common root rot caused by Bipolaris sorokiniana. *Scientific reports*, *7*(1), pp.1-15.

Zhang, S., Gan, Y. and Xu, B., 2016. Application of plant-growth-promoting fungi *Trichoderma longibrachiatum* T6 enhances tolerance of wheat to salt stress through improvement of antioxidative defense system and gene expression. *Frontiers in plant science*, *7*, p.1405.

Zhao, Y., Tian, X., Wang, F., Zhang, L., Xin, M., Hu, Z., Yao, Y., Ni, Z., Sun, Q. and Peng, H., 2017. Characterization of wheat MYB genes responsive to high temperatures. *BMC plant biology*, *17*(1), pp.1-14.

Zhu, X., Qi, L., Liu, X., Cai, S., Xu, H., Huang, R., Li, J., Wei, X. and Zhang, Z., 2014. The wheat ethylene response factor transcription factor pathogen-induced ERF1 mediates host responses to both the necrotrophic pathogen *Rhizoctonia cerealis* and freezing stresses. *Plant physiology*, *164*(3), pp.1499-1514.

Table S2. HPLC conditions for determination of phenolic compounds in rhizosphere soil solutions.

**UHPLC-MS** (identification)	**RP-HPLC** (quantitative analysis with known standards)
HPLC System: Agilent 1290 Ultra Performance Liquid Chromatography System with QExactive Plus Electrospray Mass Spectrometer (Thermo Fisher Scientific)<br><br>Column: Eclipse Plus C18, 1.8 µm 2.2 x 50 mm column (Agilent)<br><br>Eluent: 0.2% formic acid (solvent A); 0.2% formic acid in acetonitrile (solvent B)<br><br>Gradient elution: 0 -1.5 min: 0% B; 1.5 - 7 min: 0 - 15% B; 7-19 min: 15 - 75% B; 19 - 22 min 75 - 100% B; 22 - 22.1 min: 100 - 0% B; 22.1 - 23.1 min: 0% B.<br><br>Flow rate: 0.3 mL min^{-1}<br><br>Column temperature: 40°C<br><br>Detection: MS parameters: ESI positive/negative, HESI Source, Capillary Temp 360°C, Sheath gas 60, Aux gas 20, Probe Heater 380°C, resolution 70.000, NCE 20,60,110.	HPLC System: Shimadzu LC10<br><br>Column: GROM-SIL 120 ODS ST, 5 µm, 290 × 4.6 mm + 20 × 4.6 mm guard column (Grom, Herrenberg, Germany)<br><br>Eluent: 18 mM KH_2PO_4 (pH 2.49) (solvent A); methanol (solvent B)<br><br>Gradient elution: 0 - 32 min: 0 - 25% B; 32 - 33 min: 25 - 50% B; 33 - 45 min: 50 - 55% B; 45 - 50 min: 55 - 100% B; 50 - 60 min: 100% B; 60 - 70 min 100 - 0% B<br><br>Flow rate: 0.4 mL min^{-1}<br><br>Column temperature: 40°C<br><br>Detection: UV 280 nm

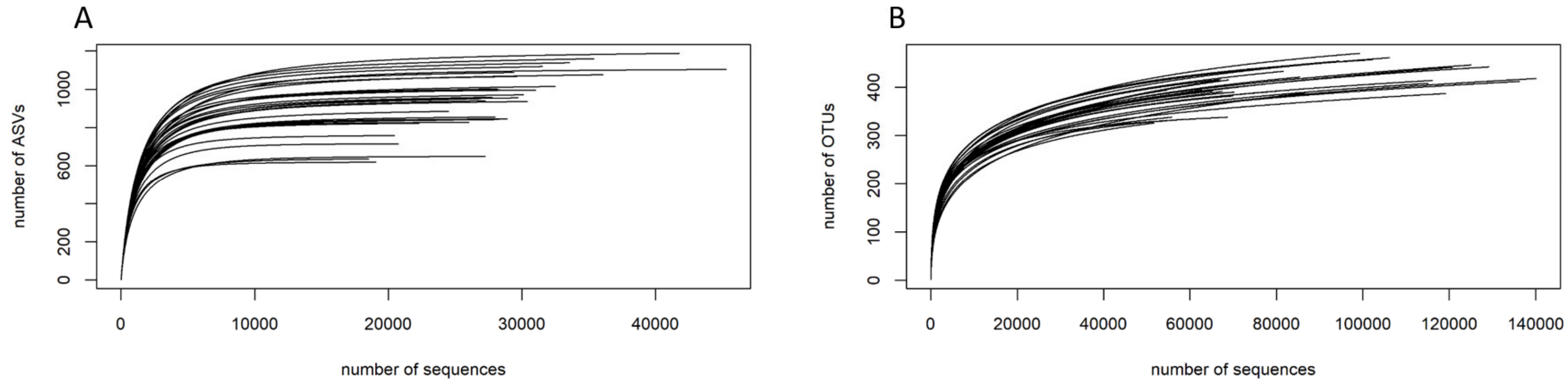

Figure S3. Rarefaction curves for A) 16S rRNA gene and B) ITS amplicon sequencing. Since all curves reached saturation, sequencing depth was sufficient to cover microbial diversity in rhizosphere and root-affected soil samples.

Table S3. Overview of Illumina barcodes and ITS2 primer combinations for each sample in root-affected soil (RA) and the rhizosphere (RH). Data are available in the European Nucleotide Archive (ENA) under PRJEB67553. MP- mouldboard plough tillage, CT- cultivator tillage, Ext- extensive N-fertilization intensity without fungicides and growth regulators, Int- intensive N-fertilization intensity with pesticides and growth regulators.

**Run**		**Barcode + Primer (forward)**	**Barcode + Primer (reverse)**
**RA soil** (ERR12207969)	**RH** (ERR12207970)		
MP-Int-1	MP-Int-1	5'-CAGGACGTGTGAATCATCGAATCTTTGAA-3'	5'-ATTACTCGTCCTCCGCTTATTGATATGC-3'
MP-Int-2	MP-Int-2		5'-TCCGGAGATCCTCCGCTTATTGATATGC-3'
MP-Int-3	MP-Int-3		5'-CGCTCATTTCCTCCGCTTATTGATATGC-3'
MP-Int-4	MP-Int-4		5'-GAGATTCCTCCTCCGCTTATTGATATGC-3'
MP-Ext-1	MP-Ext-1		5'-ATTCAGAATCCTCCGCTTATTGATATGC-3'
MP-Ext-2	MP-Ext-2		5'-GAATTCGTTCCTCCGCTTATTGATATGC-3'
MP-Ext-3	MP-Ext-3		5'-CTGAAGCTTCCTCCGCTTATTGATATGC-3'
MP-Ext-4	MP-Ext-4		5'-TAATGCGCTCCTCCGCTTATTGATATGC-3'
CT-Int-1	CT-Int-1	5'-GTACTGACGTGAATCATCGAATCTTTGAA-3'	5'-ATTACTCGTCCTCCGCTTATTGATATGC-3'
-	CT-Int-2		5'-TCCGGAGATCCTCCGCTTATTGATATGC-3'
CT-Int-3	CT-Int-3		5'-CGCTCATTTCCTCCGCTTATTGATATGC-3'
CT-Int-4	CT-Int-4		5'-GAGATTCCTCCTCCGCTTATTGATATGC-3'
CT-Ext-1	CT-Ext-1		5'-ATTCAGAATCCTCCGCTTATTGATATGC-3'
CT-Ext-2	CT-Ext-2		5'-GAATTCGTTCCTCCGCTTATTGATATGC-3'
CT-Ext-3	CT-Ext-3		5'-CTGAAGCTTCCTCCGCTTATTGATATGC-3'
CT-Ext-4	CT-Ext-4		5'-TAATGCGCTCCTCCGCTTATTGATATGC-3'

Table S4. List of plant-beneficial functions used to build the customized database based on Kyoto Encyclopedia of Genes and Genomes (KEGG) Orthology database (November 2022).

See excel file.

Table S5. Statistical analysis of the nutrient status of winter wheat (cv. Lemmy, EC 59) grown at long-term field trial Bernburg with different tillage practices (CT, cultivator tillage; MP, mouldboard plough) and N-fertilization intensities (Int, intensive N-fertilization with pesticides and growth regulators; Ext, extensive N-fertilization without fungicides and growth regulators). *P*-values from two-way ANOVA showing the effect of tillage practice and N-fertilization intensity and their interactions on nutrient levels. For metabolites with a significant interaction effect ($p<0.05$), the simple main effect was performed to test the effect of N-fertilization intensity (Fert.) at each tillage practice on metabolite level ($p<0.025$).

				**Simple-main effect**	
	**Tillage**	**Fertilization**	**Tillage x Fertilization**	**CT Fert.**	**MP Fert.**
C_{total}	0.330	0.065	0.029	0.008	0.755
N_{total}	0.100	0.003	0.028	< 0.001	0.418
P	0.526	0.568	0.019	0.159	0.038
K	0.587	0.011	0.158	-	-
Mg	0.015	< 0.001	0.205	-	-
Ca	0.003	< 0.001	0.300	-	-
S	< 0.001	< 0.001	0.436	-	-
Cu	0.003	0.338	0.756	-	-
Fe	0.056	0.064	0.464	-	-
Mn	0.282	0.805	0.736	-	-
Zn	0.597	0.382	0.901	-	-

Table S6. Statistical analysis of metabolite levels of winter wheat (cv. Lemmy, EC 59) grown in the long-term field experiment Bernburg with different tillage practices (CT, cultivator tillage; MP, mouldboard plough) and N-fertilization intensities (Int- intensive N-fertilization with pesticides and growth regulators; Ext- extensive N-fertilization without fungicides and growth regulators). *P*-values from two-way ANOVA showing the effect of tillage practice and N-fertilization intensity and their interactions on shoot, apical and basal root exudates. For metabolites with a significant interaction effect ($p<0.05$), the simple main effect was performed to test the effect of N-fertilization intensity (Fert.) at each tillage practice on metabolite level ($p<0.025$).

					**Simple-main effect**	
		**Tillage**	**Fertilization**	**Tillage x Fertilization**	**CT Fert.**	**MP Fert.**
**Shoot**	Salicylic acid	< 0.001	< 0.001	0.003	< 0.001	0.118
	Jasmonic acid	< 0.001	< 0.001	< 0.001	< 0.001	0.173
	Proline	< 0.001	< 0.001	0.469	-	-
	Phenol	< 0.001	< 0.001	< 0.001	< 0.001	0.289
	Ascorbate peroxidase	< 0.001	0.160	0.693	-	-
	Total antioxidants	< 0.001	0.216	0.369	-	-
**Apical root**	Tryptophan	< 0.001	0.431	0.469	-	-
	Asparagin	< 0.001	0.899	0.322	-	-
	Benzoic acid	< 0.001	0.007	0.026	0.628	0.002
	Caffeic acid	0.936	0.027	0.069	-	-
	Catechin*	-	-	-	-	-
	Cinnamic acid	0.622	0.043	0.082	-	-
	MBOA	< 0.001	0.161	0.110	-	-
	MeBOA	< 0.001	0.007	0.292	-	-
	Morin	0.189	0.017	0.462	-	-
	Succinic acid	< 0.001	0.392	0.138	-	-
	p-Coumaric acid	0.022	0.543	0.180	-	-
	Trehalose	< 0.001	0.015	0.032	0.003	0.778
**Basal root**	Benzoic acid	0.028	0.096	0.291	-	-
	Caffeic acid	0.020	0.827	0.064	-	-
	Catechin*	-	-	-	-	-
	Cinnamic acid	< 0.001	0.380	0.540	-	-
	MBOA	0.048	0.076	0.368	-	-
	MeBOA	0.456	0.184	0.647	-	-
	Morin	0.814	0.205	0.088	-	-
	p-Coumaric acid	< 0.001	0.482	0.482	-	-

*not statistically evaluable

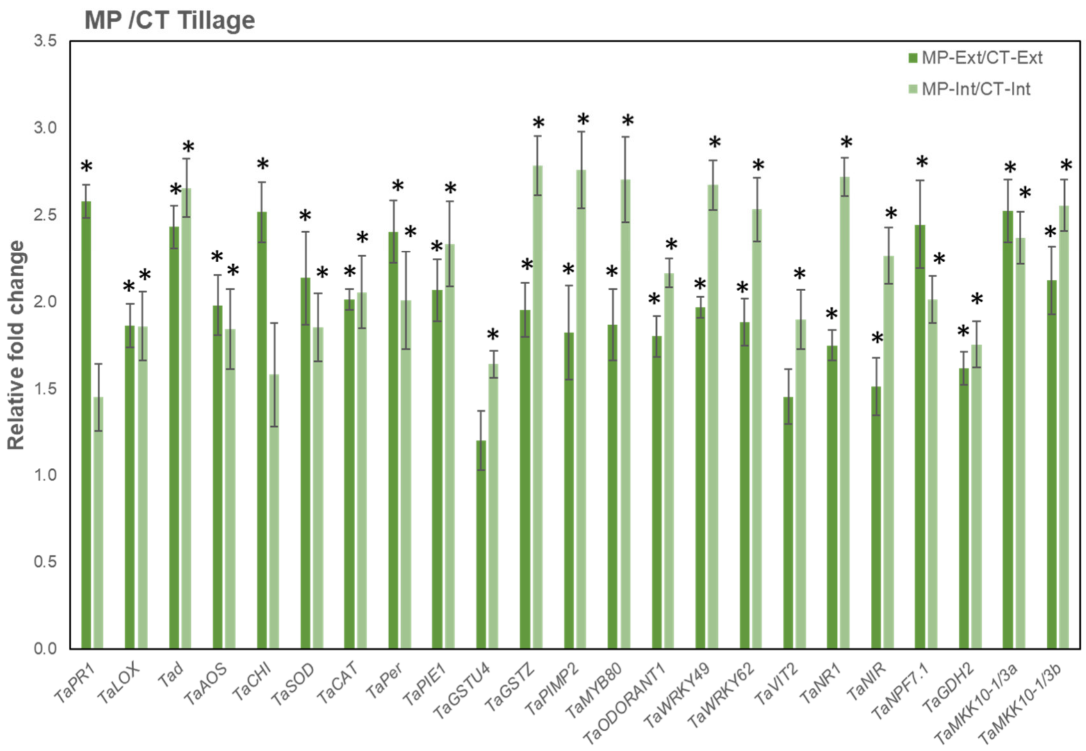

Figure S4. Log fold change in expression (ΔΔCT) of genes (23 out of 28) showing significantly enhanced expression in winter wheat plants (cv. Lemmy, EC 59) from mouldboard plough (MP) soils compared to cultivator tillage (CT) soils. Ext- extensive N-fertilization intensity without fungicides and growth regulators, Int- intensive N-fertilization intensity with pesticides and growth regulators. Asterisk indicates significant difference in ΔΔCT according to pairwise Tukey HSD (p<0.05). Error bars indicate standard error of mean (n=4).

Table S7. Effects of different long-term agricultural practices on primary and secondary metabolites in rhizosphere soil solution of apical and basal roots and root-affected soil of winter wheat (cv. Lemmy; EC 59). MP- mouldboard plough tillage, CT- cultivator tillage, Ext- extensive N-fertilization intensity without fungicides and growth regulators, Int- intensive N-fertilization intensity with pesticides and growth regulators. Values of apical and basal root-soil solution are presented as means ± standard deviation of four replicates. Root-affected soil solution values are shown as mean ± standard deviation of two replicates. Means not sharing any letters are significantly different by the Tukey-test ($p \leq 0.05$). Metabolites below the detection threshold are not shown.

	Metabolites [nmol cm^{-1} root length]							
	**MP-Int**		**MP-Ext**		**CT-Int**		**CT-Ext**	
**Apical rhizosphere soil solution**								
Asparagine	3.74 ±0.21	b	3.82 ±0.13	b	6.31 ±0.14	a	6.24 ±0.08	a
Benzoic acid	10.40 ±1.17	a	7.98 ±0.93	b	5.58 ±0.66	c	5.29 ±0.39	c
Caffeic acid	6.70 ±0.37	a	5.78 ±0.51	b	6.28 ±0.40	ab	6.17 ±0.33	ab
Catechin*	0.01 -	-	0.69 -	-	1.01 ±0.21	-	0.66 ±0.18	-
Cinnamic acid	8.84 ±1.13	a	7.63 ±0.59	b	7.98 ±0.50	ab	7.86 ±0.35	ab
MBOA	0.71 ±0.12	b	0.29 ±0.29	b	35.80 ±5.99	a	41.63 ±4.05	a
MeBOA	10.66 ±0.75	a	9.13 ±0.62	b	3.43 ±1.00	c	2.67 ±0.04	c
Morin	4.08 ±0.31	ns	3.53 ±0.31	ns	4.18 ±0.36	ns	3.87 ±0.26	ns
p-Coumaric acid	6.17 ±0.98	ns	6.47 ±0.48	ns	7.70 ±0.97	ns	6.93 ±0.35	ns
Succinic acid	1.11 ±0.14	b	1.33 ±0.13	b	1.88 ±0.22	a	1.82 ±0.22	a
Trehalose	16.00 ±4.32	c	17.75 ±6.24	c	57.48 ±11.86	b	80.00 ±9.80	a
Tryptophan	0.01 ±0.001	b	0.01 ±0.001	b	0.02 ±0.001	a	0.02 ±0.001	a
**Basal rhizosphere soil solution**								
Benzoic acid	2.03 ±1.39	ab	1.63 ±1.29	b	4.09 ±1.02	a	2.42 ±0.74	ab
Caffeic acid	0.66 ±0.45	b	0.97 ±0.26	ab	1.31 ±0.11	a	1.06 ±0.14	ab
Catechin*	0.11 ±0.20	-	0.01 -	-	0.01 -	-	0.01 -	-
Cinnamic acid	0.97 ±0.12	bc	0.95 ±0.08	c	1.24 ±0.04	a	1.16 ±0.11	ab
MBOA	0.16 ±0.25	ns	0.44 ±0.29	ns	0.04 ±0.01	ns	0.13 ±0.10	ns
MeBOA	1.76 ±0.45	ns	1.45 ±0.20	ns	1.81 ±0.24	ns	1.65 ±0.35	ns
Morin	0.53 ±0.15	ns	0.83 ±0.23	ns	0.68 ±0.19	ns	0.63 ±0.19	ns
p-Coumaric acid	0.74 ±0.10	b	0.74 ±0.06	b	0.97 ±0.04	a	0.91 ±0.09	a
**Root affected soil solution**								
Catechin*	0.10 ±0.10	-	0.96 ±0.19	-	0.69 -	-	0.76 ±0.10	-
2H-Benzoxazin*	4.25 ±0.10	-	6.19 ±0.33	-	8.90 ±1.54	-	8.83 ±1.44	-
Cinnamic acid*	1.51 ±0.27	-	1.88 ±0.52	-	1.23 ±0.17	-	1.59 ±0.69	-

*not statistically evaluable

ns, no significant difference between treatments

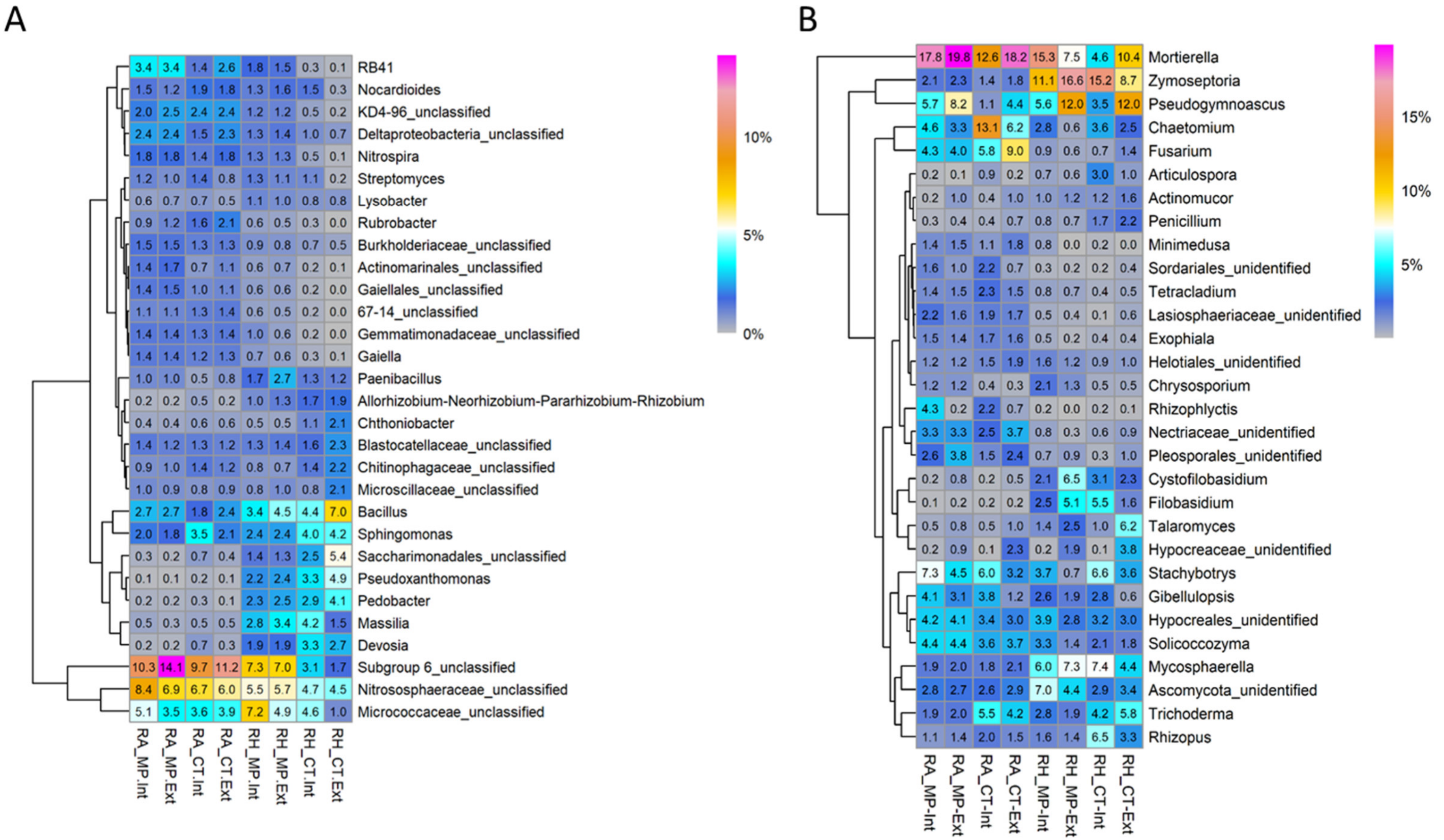

Figure S5. Heatmap showing the relative abundance distribution of the 30 most abundant A) bacterial and archaeal or B) fungal genera in root-affected soil (RA) and rhizosphere (RH) of winter wheat (cv. Lemmy, EC 59) grown in soil under different agricultural practices (MP- mouldboard plough; CT- cultivator tillage; Int- intensive N-fertilization intensity with pesticides and growth regulators; Ext- extensive N-fertilization intensity without fungicides and growth regulators). Numbers represent relative abundances.

Table S8. Bacterial and archaeal alpha-diversity in root-affected soil (RA) and rhizosphere (RH) of winter wheat (cv. Lemmy, EC 59). A) *P*-values from two-way ANOVA showing the effect of tillage practice (MP- mouldboard plough tillage, CT- cultivator tillage) and N-fertilization intensity (Ext- extensive N-fertilization without fungicides and growth regulators, Int- intensive N-fertilization with pesticides and growth regulators) and their interaction. B) Means ± standard deviation. Different letters indicate significant differences between long-term agricultural practices according to two-way ANOVA followed by Tukey's Test. **p*<0.05, ***p*<0.01, ****p*<0.001.

**A)**	**RA**			**RH**		
**Factors**	**Richness**	**Shannon**	**Pielou**	**Richness**	**Shannon**	**Pielou**
Tillage practice	0.309	0.347	<0.001***	0.212	0.159	0.674
N-Fertilization Intensity	0.001**	<0.001***	0.411	0.857	0.607	0.470
Tillage practice × N-Fertilization Intensity	0.945	0.480	0.008**	0.743	0.939	0.360

**B)**	**RA**			**RH**		
**Treatments**	**Richness**	**Shannon**	**Pielou**	**Richness**	**Shannon**	**Pielou**
MP-Int	910.1 ± 51.8 b	6.22 ± 0.05 b	0.91 ± 0 c	948.2 ± 136.7 a	6.19 ± 0.08 a	0.90 ± 0.01 a
MP-Ext	1073.1 ± 79.1 a	6.41 ± 0.08 a	0.92 ± 0 bc	904.9 ± 184.7 a	6.15 ± 0.18 a	0.91 ± 0 a
CT-Int	854.3 ± 118.6 b	6.27 ± 0.09 ab	0.93 ± 0.01 a	810.6 ± 197.3 a	6.06 ± 0.1 a	0.91 ± 0.02 a
CT-Ext	1022.7 ± 44.2 ab	6.41 ± 0.04 a	0.93 ± 0 ab	823.3 ± 138.5 a	6.00 ± 0.29 a	0.90 ± 0.02 a

Table S9. Fungal alpha-diversity in root-affected soil (RA) and rhizosphere (RH) of winter wheat (cv. Lemmy, EC 59). A) *P*-values from two-way-ANOVA showing the effect of tillage practice (MP- mouldboard plough tillage, CT- cultivator tillage) and N-fertilization intensity (Ext- extensive N-fertilization without fungicides and growth regulators, Int- intensive N-fertilization with pesticides and growth regulators) and their interaction. B) Means ± standard deviation. Different letters indicate significant differences between long-term agricultural treatments by two-way ANOVA followed by Tukey's Test. If ANOVA assumptions failed, data were transformed by Tukey's Ladder of Power (†). *$p<0.05$, **$p<0.01$, ***$p<0.001$.

**A)**	**RA**			**RH**		
**Factors**	**Richness**	**Shannon**	**Pielou**	**Richness**	**Shannon**	**Pielou**
Tillage practice	0.0096**	0.9240	0.1197	0.0960	0.766	0.617
N-Fertilization Intensity	0.0570	0.0260*	0.1785	0.0089**	0.331	0.157
Tillage practice × N-Fertilization Intensity	0.4540	0.0085**	0.0138*	0.1440	0.053	0.050

**B)**	**RA**			**RH**		
**Treatments**	**Richness**	**Shannon**	**Pielou**	**Richness**	**Shannon**	**Pielou**
MP-Int	360.0 ± 19.5 b	4.18 ± 0.06 ab	0.71 ± 0.01 a	351.3 ± 25.3 b	4.05 ± 0.22 a	0.69 ± 0.03 a
MP-Ext	371.5 ± 9.8 ab	4.17 ± 0.09 ab	0.70 ± 0.02 ab	368.5 ± 18.3 ab	3.64 ± 0.39 a	0.62 ± 0.06 a
CT-Int	382.3 ± 33.5 ab	4.07 ± 0.06 b	0.69 ± 0.01 b	353.3 ± 27.1 b	3.74 ± 0.17 a	0.64 ± 0.02 a
CT-Ext	409.0 ± 8.2 a	4.27 ± 0.03 a	0.71 ± 0 a	391.3 ± 5.0 a	3.88 ± 0.20 a	0.65 ± 0.03 a

Table S10. Bacterial/Archaeal species (ASV) with differential relative abundance in the rhizosphere of winter wheat (cv. Lemmy, EC 59) grown in soil under MP- mouldboard plough tillage and CT- cultivator tillage practice (FDR<0.05). Only discriminative species with mean >0.1% are displayed. Significantly enriched taxa are marked in bold. Mean ± standard deviation is shown (n=8).

Phylum	Class	Order	Family	Genus	ASV	MP	CT
Acidobacteria	Blastocatellia (Subgroup 4)	Pyrinomonadales	*Pyrinomonadaceae*	*RB41*	ASV28818	**0.2 ± 0.1**	0 ± 0
Acidobacteria	Subgroup 6	Subgroup 6_unclassified	*Subgroup 6_unclassified*	*Subgroup 6_unclassified*	ASV31872	**0.2 ± 0**	0 ± 0
Acidobacteria	Subgroup 6	uncultivated soil bacterium clone C028	*uncultivated soil bacterium clone C028_unclassified*	*uncultivated soil bacterium clone C028_unclassified*	ASV6835	**0.2 ± 0.1**	0 ± 0
Acidobacteria	Blastocatellia (Subgroup 4)	Pyrinomonadales	*Pyrinomonadaceae*	*RB41*	ASV36824	**0.1 ± 0.1**	0 ± 0
Acidobacteria	Subgroup 6	Subgroup 6_unclassified	*Subgroup 6_unclassified*	*Subgroup 6_unclassified*	ASV17915	**0.1 ± 0.1**	0 ± 0
Acidobacteria	Blastocatellia (Subgroup 4)	Pyrinomonadales	*Pyrinomonadaceae*	*RB41*	ASV4136	**0.5 ± 0.2**	0 ± 0.1
Acidobacteria	Subgroup 6	Subgroup 6_unclassified	*Subgroup 6_unclassified*	*Subgroup 6_unclassified*	ASV47414	**0.3 ± 0.1**	0 ± 0.1
Acidobacteria	Subgroup 6	uncultured bacterium gp6	*uncultured bacterium gp6_unclassified*	*uncultured bacterium gp6_unclassified*	ASV19064	**0.1 ± 0**	0 ± 0
Actinobacteria	Actinobacteria	Micrococcales	*Micrococcaceae*	*Micrococcaceae_unclassified*	ASV49496	**2.3 ± 0.8**	0.9 ± 0.7
Actinobacteria	Actinobacteria	Micrococcales	*Microbacteriaceae*	*Agromyces*	ASV660	**0.7 ± 0.1**	0.2 ± 0.2
Actinobacteria	Actinobacteria	Micrococcales	*Micrococcaceae*	*Micrococcaceae_unclassified*	ASV16162	**0.5 ± 0.2**	0 ± 0.1
Actinobacteria	Actinobacteria	Streptomycetales	*Streptomycetaceae*	*Streptomyces*	ASV43643	**0.1 ± 0.1**	0 ± 0
Actinobacteria	Actinobacteria	Frankiales	*Geodermatophilaceae*	*Blastococcus*	ASV8669	**0.1 ± 0.1**	0 ± 0
Actinobacteria	Actinobacteria	Micromonosporales	*Micromonosporaceae*	*Catellatospora*	ASV46965	**0.1 ± 0**	0 ± 0
Actinobacteria	Acidimicrobiia	Actinomarinales	*Actinomarinales_unclassified*	*Actinomarinales_unclassified*	ASV45546	**0.1 ± 0.1**	0 ± 0
Bacteroidetes	Bacteroidia	Chitinophagales	*Chitinophagaceae*	*Terrimonas*	ASV11972	**0.7 ± 0.2**	0.4 ± 0.2
Bacteroidetes	Bacteroidia	Sphingobacteriales	*Sphingobacteriaceae*	*Pedobacter*	ASV24586	**0.3 ± 0.2**	0 ± 0.1
Bacteroidetes	Bacteroidia	Flavobacteriales	*Flavobacteriaceae*	*Flavobacterium*	ASV31878	**0.1 ± 0.1**	0 ± 0
Chloroflexi	KD4-96	KD4-96_unclassified	*KD4-96_unclassified*	*KD4-96_unclassified*	ASV19302	**0.2 ± 0**	0 ± 0
Chloroflexi	Dehalococcoidia	S085	*S085_unclassified*	*S085_unclassified*	ASV6612	**0.1 ± 0.1**	0 ± 0
Chloroflexi	KD4-96	KD4-96_unclassified	*KD4-96_unclassified*	*KD4-96_unclassified*	ASV34550	**0.1 ± 0.2**	0 ± 0
Firmicutes	Bacilli	Bacillales	*Paenibacillaceae*	*Paenibacillus*	ASV2746	**0.4 ± 0.1**	0.2 ± 0.1
Firmicutes	Bacilli	Bacillales	*Paenibacillaceae*	*Cohnella*	ASV41641	**0.2 ± 0.1**	0.1 ± 0
Nitrospirae	Nitrospira	Nitrospirales	*Nitrospiraceae*	*Nitrospira*	ASV33194	**0.6 ± 0.1**	0.2 ± 0.2
Nitrospirae	Nitrospira	Nitrospirales	*Nitrospiraceae*	*Nitrospira*	ASV35563	**0.5 ± 0.1**	0 ± 0.1

Patescibacteria	Saccharimonadia	Saccharimonadales	*Saccharimonadaceae*	*Saccharimonadaceae_unclassified*	ASV10175	**0.3 ± 0.1**	0 ± 0.1
Patescibacteria	Saccharimonadia	Saccharimonadales	*Saccharimonadales_unclassified*	*Saccharimonadales_unclassified*	ASV9848	**0.1 ± 0.1**	0 ± 0
Proteobacteria	Gammaproteobacteria	Betaproteobacteriales	*Burkholderiaceae*	*Massilia*	ASV41323	**0.2 ± 0.2**	0 ± 0
Proteobacteria	Gammaproteobacteria	Xanthomonadales	*Xanthomonadaceae*	*Lysobacter*	ASV37269	**0.1 ± 0.1**	0 ± 0
Proteobacteria	Gammaproteobacteria	Betaproteobacteriales	*Nitrosomonadaceae*	*MND1*	ASV4972	**0.1 ± 0**	0 ± 0
Thaumarchaeota	Nitrososphaeria	Nitrososphaerales	*Nitrososphaeraceae*	*Nitrososphaeraceae_unclassified*	ASV42131	**1.4 ± 0.5**	1 ± 0.3
Thaumarchaeota	Nitrososphaeria	Nitrososphaerales	*Nitrososphaeraceae*	*Nitrososphaeraceae_unclassified*	ASV35975	**0.4 ± 0.1**	0 ± 0.1
Acidobacteria	Blastocatellia (Subgroup 4)	Blastocatellales	*Blastocatellaceae*	*Blastocatellaceae_unclassified*	ASV14058	0 ± 0	**0.1 ± 0.1**
Actinobacteria	Actinobacteria	Micrococcales	*Microbacteriaceae*	*Microbacteriaceae_unclassified*	ASV37410	0 ± 0	**0.2 ± 0.1**
Actinobacteria	Actinobacteria	Micrococcales	*Microbacteriaceae*	*Microbacteriaceae_unclassified*	ASV10235	0 ± 0	**0.1 ± 0.1**
Bacteroidetes	Bacteroidia	Cytophagales	*Microscillaceae*	*Chryseolinea*	ASV33311	0 ± 0	**0.1 ± 0.1**
Bacteroidetes	Bacteroidia	Cytophagales	*Spirosomaceae*	*Dyadobacter*	ASV20945	0 ± 0	**0.2 ± 0.2**
Chloroflexi	Anaerolineae	SBR1031	*A4b*	*A4b_unclassified*	ASV3082	0 ± 0	**0.2 ± 0.1**
Firmicutes	Bacilli	Bacillales	*Bacillaceae*	*Bacillus*	ASV13035	0.2 ± 0.1	**0.5 ± 0.2**
Firmicutes	Bacilli	Bacillales	*Bacillaceae*	*Bacillus*	ASV47072	0.2 ± 0.1	**1 ± 0.4**
Firmicutes	Bacilli	Bacillales	*Bacillaceae*	*Bacillus*	ASV10264	0.2 ± 0.1	**0.7 ± 0.3**
Patescibacteria	Saccharimonadia	Saccharimonadales	*Saccharimonadaceae*	*Saccharimonadaceae_unclassified*	ASV36575	0 ± 0	**0.3 ± 0.2**
Patescibacteria	Saccharimonadia	Saccharimonadales	*Saccharimonadaceae*	*Saccharimonadaceae_unclassified*	ASV51676	0 ± 0	**0.5 ± 0.9**
Patescibacteria	Saccharimonadia	Saccharimonadales	*Saccharimonadales_unclassified*	*Saccharimonadales_unclassified*	ASV37232	0 ± 0	**0.1 ± 0.2**
Patescibacteria	Saccharimonadia	Saccharimonadales	*Saccharimonadales_unclassified*	*Saccharimonadales_unclassified*	ASV21430	0 ± 0	**0.1 ± 0.1**
Patescibacteria	Saccharimonadia	Saccharimonadales	*Saccharimonadales_unclassified*	*Saccharimonadales_unclassified*	ASV47124	0 ± 0	**0.2 ± 0.4**
Proteobacteria	Alphaproteobacteria	Sphingomonadales	*Sphingomonadaceae*	*Sphingomonas*	ASV40734	0.2 ± 0.1	**0.6 ± 0.2**
Proteobacteria	Alphaproteobacteria	Sphingomonadales	*Sphingomonadaceae*	*Sphingomonas*	ASV7204	0 ± 0	**0.3 ± 0.3**
Proteobacteria	Alphaproteobacteria	Rhizobiales	*Rhizobiaceae*	*Mesorhizobium*	ASV47384	0 ± 0	**0.1 ± 0.1**
Proteobacteria	Alphaproteobacteria	Sphingomonadales	*Sphingomonadaceae*	*Sphingomonas*	ASV15752	0 ± 0	**0.2 ± 0.3**
Proteobacteria	Alphaproteobacteria	Rhizobiales	*Devosiaceae*	*Devosia*	ASV30746	0 ± 0	**0.1 ± 0.2**
Proteobacteria	Alphaproteobacteria	Rhizobiales	*Devosiaceae*	*Devosia*	ASV38130	0 ± 0	**0.1 ± 0.2**
Proteobacteria	Alphaproteobacteria	Rhizobiales	*Devosiaceae*	*Devosia*	ASV1636	0.2 ± 0.1	**0.7 ± 0.3**
Proteobacteria	Gammaproteobacteria	Betaproteobacteriales	*Methylophilaceae*	*Methylotenera*	ASV11982	0.1 ± 0.1	**0.4 ± 0.3**
Proteobacteria	Gammaproteobacteria	Betaproteobacteriales	*Burkholderiaceae*	*Caenimonas*	ASV21850	0 ± 0	**0.2 ± 0.1**

Proteobacteria	Gammaproteobacteria	Xanthomonadales	*Xanthomonadaceae*	*Pseudoxanthomonas*	ASV36003	0 ± 0	**0.2 ± 0.2**
Proteobacteria	Gammaproteobacteria	Xanthomonadales	*Xanthomonadaceae*	*Pseudoxanthomonas*	ASV9333	0 ± 0	**0.1 ± 0.1**
Proteobacteria	Gammaproteobacteria	Xanthomonadales	*Xanthomonadaceae*	*Pseudoxanthomonas*	ASV48785	1.8 ± 0.3	**3.2 ± 1.8**
Thaumarchaeota	Nitrososphaeria	Nitrososphaerales	*Nitrososphaeraceae*	*Nitrososphaeraceae_unclassified*	ASV24360	0.1 ± 0	**0.6 ± 0.2**
Verrucomicrobia	Verrucomicrobiae	Chthoniobacterales	*Chthoniobacteraceae*	*Chthoniobacter*	ASV39202	0.1 ± 0.1	**0.3 ± 0.2**

Table S11. Fungal species (ASVs) with differential relative abundance in the rhizosphere of winter wheat (cv. Lemmy, EC 59) grown in soil under MP- mouldboard plough tillage or CT- cultivator tillage practice (FDR<0.05). Only discriminative species with mean >0.1% are displayed. Significantly enriched taxa are marked in bold. Mean ± standard deviation is shown (n=8), unident. - unidentified, inc. sed. – incertae sedis.

Phylum	Class	Order	Family	Genus	ASV	MP	CT
Ascomycota	Ascomycota_unident.	Ascomycota_unident.	*Ascomycota_unident.*	*Ascomycota_unident.*	*Ascomycota sp*	**2.0 ± 1.0**	0.3 ± 0.1
Ascomycota	Eurotiomycetes	Eurotiales	*Trichocomaceae*	*Talaromyces*	*Talaromyces sayulitensis*	**0.2 ± 0.3**	0 ± 0
Ascomycota	Eurotiomycetes	Onygenales	*Onygenales _inc. sed.*	*Chrysosporium*	*Chrysosporium lobatum*	**1.3 ± 0.9**	0.5 ± 0.3
Ascomycota	Eurotiomycetes	Onygenales	*Onygenales _inc. sed.*	*Chrysosporium*	*Chrysosporium pseudomerdarium*	**0.3 ± 0.3**	0 ± 0
Ascomycota	Eurotiomycetes	Onygenales	*Onygenales_unident.*	*Onygenales_unident.*	*Onygenales sp*	**0.1 ± 0.1**	0 ± 0
Ascomycota	Leotiomycetes	Helotiales	*Helotiaceae*	*Scytalidium*	*Scytalidium circinatum*	**0.2 ± 0.2**	0 ± 0
Ascomycota	Leotiomycetes	Leotiomycetes_unident.	*Leotiomycetes_unident.*	*Leotiomycetes_unident.*	*Leotiomycetes sp*	**0.2 ± 0.2**	0 ± 0
Ascomycota	Leotiomycetes	Thelebolales	*Pseudeurotiaceae*	*Pseudogymnoascus*	*Pseudogymnoascus pannorum*	**1.4 ± 0.7**	0.2 ± 0.1
Ascomycota	Pezizomycetes	Pezizales	*Pyronemataceae*	*Pyronemataceae_unident.*	*Pyronemataceae sp*	**0.3 ± 0.2**	0.1 ± 0.1
Ascomycota	Sordariomycetes	Hypocreales	*Hypocreaceae*	*Trichoderma*	*Trichoderma barbatum*	**0.3 ± 0.2**	0 ± 0
Ascomycota	Sordariomycetes	Hypocreales	*Hypocreales _inc. sed.*	*Acremonium*	*Acremonium persicinum*	**0.6 ± 0.2**	0.1 ± 0
Ascomycota	Sordariomycetes	Sordariales	*Lasiosphaeriaceae*	*Lasiosphaeriaceae_unident.*	*Lasiosphaeriaceae sp*	**0.2 ± 0.1**	0 ± 0
Basidiomycota	Agaricomycetes	Agaricales	*Strophariaceae*	*Strophariaceae_unident.*	*Strophariaceae sp*	**1.2 ± 2.1**	0.1 ± 0.1
Basidiomycota	Agaricomycetes	Cantharellales	*Ceratobasidiaceae*	*Ceratobasidiaceae_unident.*	*Ceratobasidiaceae sp*	**0.2 ± 0.2**	0 ± 0
Basidiomycota	Cystobasidiomycetes	Cystobasidiomycetes _inc. sed.	*Symmetrosporaceae*	*Symmetrospora*	*Symmetrospora coprosmae*	**0.2 ± 0.1**	0.1 ± 0
Basidiomycota	Exobasidiomycetes	Entylomatales	*Entylomatales_unident.*	*Entylomatales_unident.*	*Entylomatales sp*	**0.2 ± 0.3**	0 ± 0
Basidiomycota	Microbotryomycetes	Sporidiobolales	*Sporidiobolaceae*	*Sporidiobolaceae_unident.*	*Sporidiobolaceae sp*	**1.3 ± 0.7**	0.4 ± 0.3
Basidiomycota	Tremellomycetes	Filobasidiales	*Filobasidiaceae*	*Filobasidium*	*Filobasidium magnum*	**0.1 ± 0.2**	0 ± 0
Chytridiomycota	Spizellomycetes	Spizellomycetales	*Powellomycetaceae*	*Powellomyces*	*Powellomyces hirtus*	**0.1 ± 0.1**	0 ± 0
Mortierellomycota	Mortierellomycetes	Mortierellales	*Mortierellaceae*	*Mortierella*	*Mortierella fimbricystis*	**0.9 ± 0.6**	0.1 ± 0
Mortierellomycota	Mortierellomycetes	Mortierellales	*Mortierellaceae*	*Mortierella*	*Mortierella antarctica*	**0.5 ± 0.4**	0.1 ± 0
Mortierellomycota	Mortierellomycetes	Mortierellales	*Mortierellaceae*	*Mortierella*	*Mortierella gamsii*	**0.4 ± 0.5**	0.1 ± 0.1
Mortierellomycota	Mortierellomycetes	Mortierellales	*Mortierellaceae*	*Mortierella*	*Mortierella amoeboidea*	**0.1 ± 0.3**	0 ± 0
Olpidiomycota	Olpidiomycetes	Olpidiales	*Olpidiaceae*	*Olpidium*	*Olpidium brassicae*	**1.0 ± 1.5**	0 ± 0
Ascomycota	Ascomycota_unident.	Ascomycota_unident.	*Ascomycota_unident.*	*Ascomycota_unident.*	*Ascomycota sp*	0 ± 0	**0.1 ± 0.1**

Ascomycota	Dothideomycetes	Pleosporales	*Cucurbitariaceae*	*Pyrenochaetopsis*	*Pyrenochaetopsis leptospora*	0 ± 0	**0.2 ± 0.1**
Ascomycota	Dothideomycetes	Pleosporales	*Leptosphaeriaceae*	*Leptosphaeriaceae_unident.*	*Leptosphaeriaceae sp*	0.2 ± 0.2	**1.2 ± 0.6**
Ascomycota	Dothideomycetes	Pleosporales	*Leptosphaeriaceae*	*Leptosphaeria*	*Leptosphaeria maculans*	0 ± 0	**0.2 ± 0.2**
Ascomycota	Dothideomycetes	Pleosporales	*Pleosporales_unident.*	*Pleosporales_unident.*	*Pleosporales sp*	0 ± 0	**0.2 ± 0.3**
Ascomycota	Eurotiomycetes	Eurotiales	*Aspergillaceae*	*Penicillium*	*Penicillium aethiopicum*	0.6 ± 0.3	**1.7 ± 0.5**
Ascomycota	Leotiomycetes	Helotiales	*Helotiaceae*	*Articulospora*	*Articulospora sp*	0.7 ± 0.6	**2.0 ± 1.4**
Ascomycota	Leotiomycetes	Helotiales	*Helotiaceae*	*Hymenoscyphus*	*Hymenoscyphus menthae*	0 ± 0	**0.1 ± 0.3**
Ascomycota	Pezizomycetes	Pezizales	*Pezizales_unident.*	*Pezizales_unident.*	*Pezizales sp*	0 ± 0	**0.1 ± 0.2**
Ascomycota	Sordariomycetes	Glomerellales	*Plectosphaerellaceae*	*Plectosphaerella*	*Plectosphaerella alismatis*	0 ± 0	**0.3 ± 0.2**
Ascomycota	Sordariomycetes	Hypocreales	*Hypocreaceae*	*Trichoderma*	*Trichoderma petersenii*	0.4 ± 0.2	**2.0 ± 1.8**
Ascomycota	Sordariomycetes	Hypocreales	*Hypocreaceae*	*Trichoderma*	*Trichoderma piluliferum*	0.5 ± 0.4	**1.8 ± 0.8**
Ascomycota	Sordariomycetes	Hypocreales	*Hypocreales _inc. sed.*	*Sarocladium*	*Sarocladium bactrocephalum*	0 ± 0	**0.3 ± 0.4**
Ascomycota	Sordariomycetes	Hypocreales	*Nectriaceae*	*Stephanonectria*	*Stephanonectria keithii*	0 ± 0	**0.1 ± 0.1**
Ascomycota	Sordariomycetes	Hypocreales	*Nectriaceae*	*Fusarium*	*Fusarium sp*	0.2 ± 0.2	**0.6 ± 0.4**
Ascomycota	Sordariomycetes	Hypocreales	*Nectriaceae*	*Nectriaceae_unident.*	*Nectriaceae sp*	0.1 ± 0.1	**0.4 ± 0.2**
Ascomycota	Sordariomycetes	Hypocreales	*Stachybotryaceae*	*Stachybotrys*	*Stachybotrys chartarum*	2.2 ± 1.9	**5.1 ± 2.5**
Ascomycota	Sordariomycetes	Hypocreales	*Stachybotryaceae*	*Stachybotryaceae_unident.*	*Stachybotryaceae sp*	0 ± 0	**0.3 ± 0.5**
Ascomycota	Sordariomycetes	Sordariales	*Chaetomiaceae*	*Chaetomium*	*Chaetomium sp*	1.6 ± 2.3	**3.0 ± 0.9**
Ascomycota	Sordariomycetes	Sordariales	*Lasiosphaeriaceae*	*Schizothecium*	*Schizothecium carpinicola*	0 ± 0	**0.1 ± 0.1**
Basidiomycota	Tremellomycetes	Cystofilobasidiales	*Mrakiaceae*	*Tausonia*	*Tausonia pullulans*	0.3 ± 0.2	**0.7 ± 0.4**
Basidiomycota	Tremellomycetes	Tremellales	*Bulleribasidiaceae*	*Vishniacozyma*	*Vishniacozyma dimennae*	0 ± 0	**0.1 ± 0.1**
Chytridiomycota	Rhizophlyctidomycetes	Rhizophlyctidales	*Rhizophlyctidaceae*	*Rhizophlyctis*	*Rhizophlyctis rosea*	0 ± 0	**0.1 ± 0.1**
Mucoromycota	Mucoromycetes	Mucorales	*Mucoraceae*	*Mucor*	*Mucor circinelloides*	0 ± 0	**0.1 ± 0.1**
Mucoromycota	Mucoromycetes	Mucorales	*Rhizopodaceae*	*Rhizopus*	*Rhizopus arrhizus*	1.5 ± 0.8	**4.9 ± 2.1**

Table S12. Fungal species (ASVs) with differential relative abundance in the rhizosphere of winter wheat (cv. Lemmy, EC 59) grown in soil under intensive (Int) or extensive (Ext) N-fertilization intensity (FDR<0.05) in root window setup. Only discriminative species with mean >0.1% are displayed. Significantly enriched taxa are marked in bold. Mean ± standard deviation is shown (n=8), unident. - unidentified, inc. sed. – incertae sedis.

Phylum	Class	Order	Family	Genus	ASV	Int	Ext
Ascomycota	Ascomycota_unident.	Ascomycota_unident.	*Ascomycota_unident.*	*Ascomycota_unident.*	*Ascomycota sp*	**1.0 ± 0.6**	0.2 ± 0.1
Ascomycota	Sordariomycetes	Glomerellales	*Plectosphaerellaceae*	*Plectosphaerella*	*Plectosphaerella cucumerina*	**0.4 ± 0.3**	0.1 ± 0
Ascomycota	Sordariomycetes	Hypocreales	*Clavicipitaceae*	*Metarhizium*	*Metarhizium carneum*	**0.4 ± 0.5**	0.1 ± 0.1
Ascomycota	Sordariomycetes	Hypocreales	*Hypocreales_inc. sed.*	*Acremonium*	*Acremonium rutilum*	**0.3 ± 0.2**	0 ± 0
Basidiomycota	Agaricomycetes	Cantharellales	*Cantharellales_inc. sed.*	*Minimedusa*	*Minimedusa polyspora*	**0.5 ± 0.7**	0 ± 0
Fungi_unident.	Fungi_unident.	Fungi_unident.	*Fungi_unident.*	*Fungi_unident.*	*Fungi sp*	**0.1 ± 0.1**	0 ± 0
Ascomycota	Dothideomycetes	Pleosporales	*Pleosporales_unident.*	*Pleosporales_unident.*	*Pleosporales sp*	0 ± 0	**0.2 ± 0.3**
Ascomycota	Eurotiomycetes	Eurotiales	*Trichocomaceae*	*Talaromyces*	*Talaromyces veerkampii*	0.5 ± 0.2	**3.6 ± 4.8**
Ascomycota	Eurotiomycetes	Eurotiales	*Trichocomaceae*	*Talaromyces*	*Talaromyces sayulitensis*	0 ± 0	**0.2 ± 0.3**
Ascomycota	Leotiomycetes	Thelebolales	*Pseudeurotiaceae*	*Pseudogymnoascus*	*Pseudogymnoascus appendiculatus*	3.4 ± 1.2	**11.5 ± 8.0**
Ascomycota	Pezizomycetes	Pezizales	*Pezizales_unident.*	*Pezizales_unident.*	*Pezizales sp*	0 ± 0	**0.1 ± 0.2**
Ascomycota	Sordariomycetes	Hypocreales	*Hypocreaceae*	*Hypocreaceae_unident.*	*Hypocreaceae sp*	0.2 ± 0.1	**2.9 ± 1.7**
Ascomycota	Sordariomycetes	Hypocreales	*Nectriaceae*	*Fusarium*	*Fusarium sp*	0 ± 0	**0.4 ± 0.3**
Basidiomycota	Agaricomycetes	Agaricales	*Bolbitiaceae*	*Conocybe*	*Conocybe cylindracea*	0.1 ± 0.1	**0.4 ± 0.6**
Basidiomycota	Cystobasidiomycetes	Cystobasidiomycetes_inc. sed.	*Symmetrosporaceae*	*Symmetrospora*	*Symmetrospora coprosmae*	0.1 ± 0	**0.2 ± 0.1**
Basidiomycota	Exobasidiomycetes	Entylomatales	*Entylomatales_unident.*	*Entylomatales_unident.*	*Entylomatales sp*	0 ± 0	**0.2 ± 0.4**
Basidiomycota	Tremellomycetes	Filobasidiales	*Filobasidiaceae*	*Filobasidium*	*Filobasidium oeirense*	0.3 ± 0.2	**1.0 ± 0.7**
Basidiomycota	Tremellomycetes	Filobasidiales	*Filobasidiaceae*	*Filobasidium*	*Filobasidium magnum*	0 ± 0	**0.2 ± 0.2**
Mortierellomycota	Mortierellomycetes	Mortierellales	*Mortierellaceae*	*Mortierella*	*Mortierella alpina*	1.2 ± 0.9	**2.4 ± 1.5**
Olpidiomycota	Olpidiomycetes	Olpidiales	*Olpidiaceae*	*Olpidium*	*Olpidium brassicae*	0 ± 0	**1.0 ± 1.5**

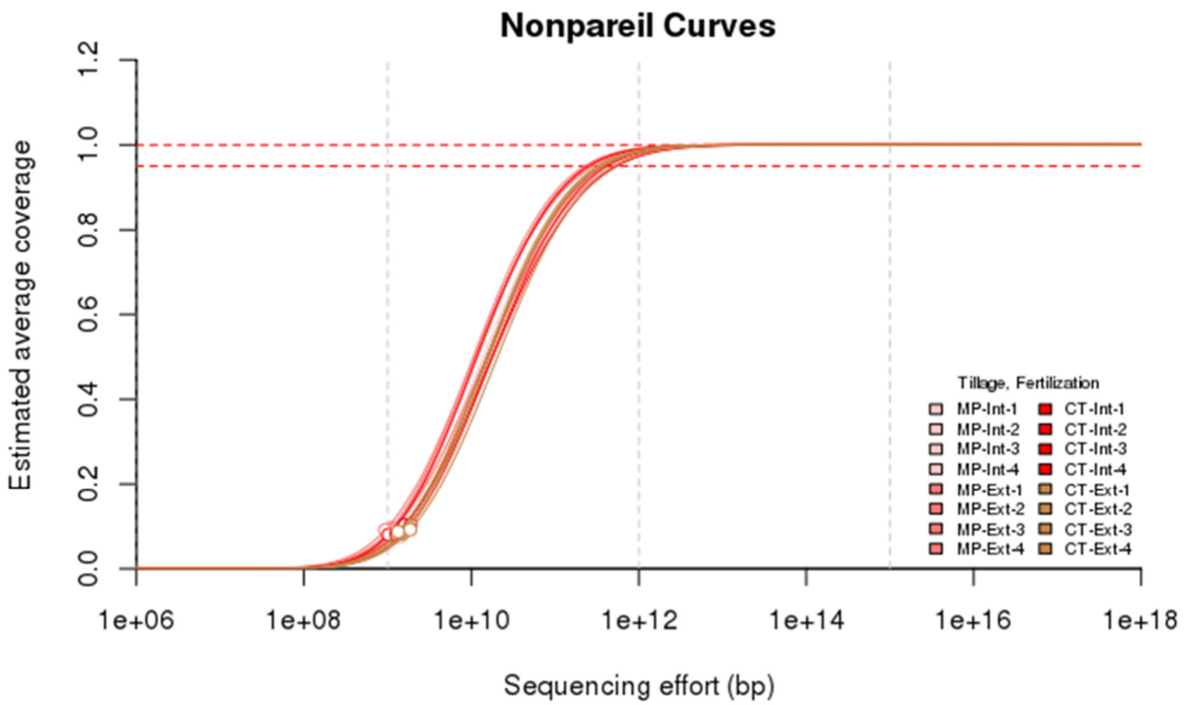

Figure S7. Nonpareil curves generated from shotgun metagenome sequencing showing the estimated coverage of the diversity per sample. Estimated average coverage is given in percent with 0.2 = 20%.

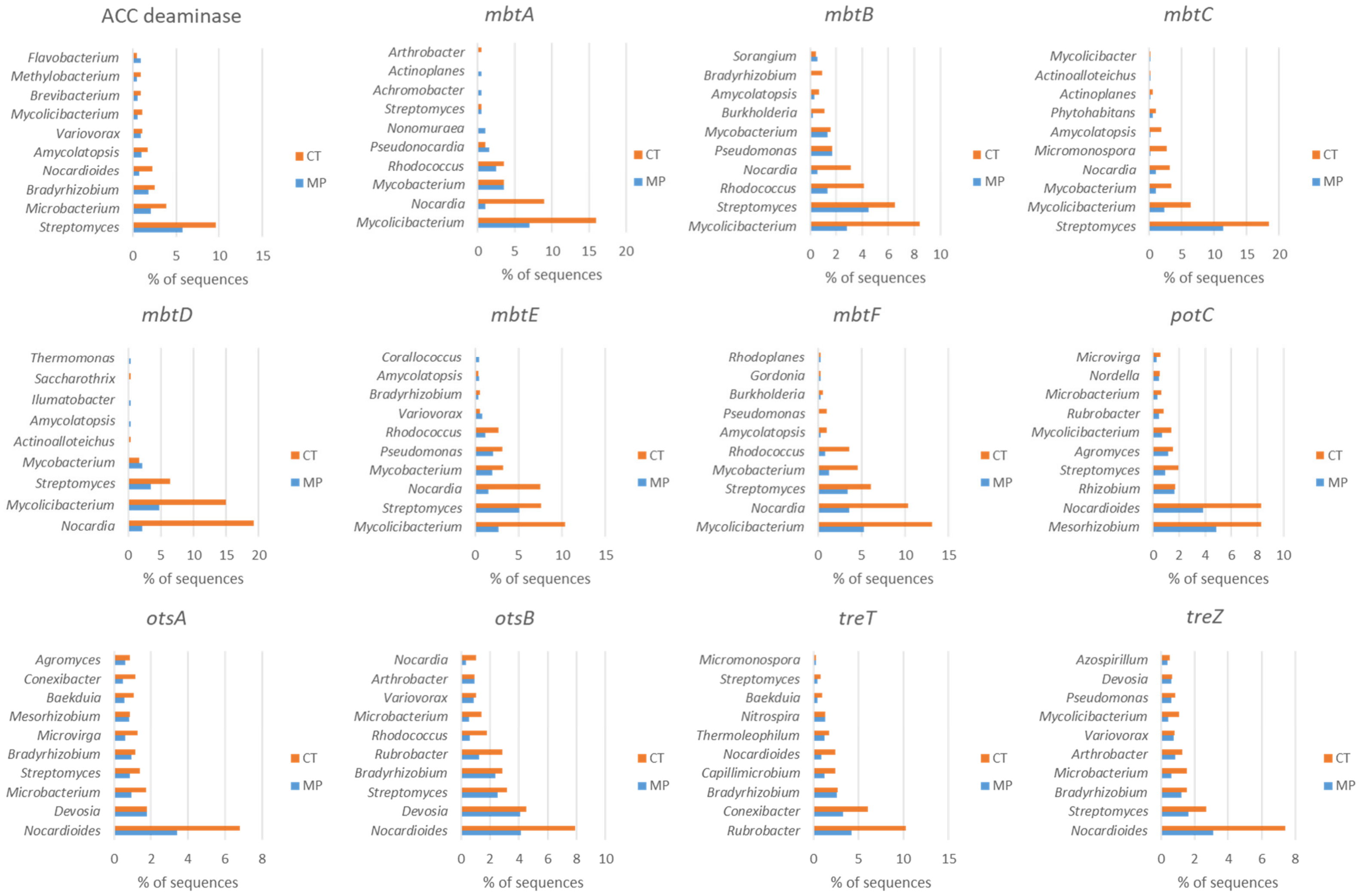

Figure S8. Taxonomic classification of functional genes, which were enriched in the rhizosphere of winter wheat (cv. Lemmy, EC 5) grown in soil under cultivator tillage (CT) compared to mouldboard plough (MP) practice, to bacterial genera. Presented are the ten most abundant genera in percent (%).

Table S13. Tentative phylogenetic affiliation of bacterial/archaeal ASVs with differential relative abundance (mean >0.5%) in the rhizosphere of winter wheat (cv. Lemmy, EC 59) grown in MP vs. CT soils (FDR < 0.05; Table 6) using BLAST-NCBI database (only type material; last access 15th Aug 2023). These responder ASVs were used for correlation analysis (Fig. 4).

ASV	ASV_code	Closest BLAST hit	E-value	% Identity	Accession No.
ASV4136	154b2b2a745651308f22aa62f5588a29	*Brevitalea aridisoli*	1.00E-177	93.98%	NR_151987.1
ASV49496	f2f1d769ca62216f2e97e5d0a34fe83e	*Arthrobacter ulcerisalmonis*	0	100.00%	NR_180330.1
ASV660	03488ba0e533f5a3bdfaacf062cebbc8	*Agromyces ramosus*	0	100.00%	NR_026165.1
ASV16162	4fb99d087105de2a392094534160d00f	*Arthrobacter ulcerisalmonis*	0	99.75%	NR_180330.1
ASV11972	3b4d1ccea11192cfca9f57f04498cc7b	*Terrimonas soli*	0	97.79%	NR_159891.1
ASV33194	a31e927c29b211c2217ceab4811ccd93	*Nitrospira japonica*	0	99.01%	NR_114396.1
ASV35563	aeb2943e3abd311274dd041ee2a7fb6e	*Nitrospira moscoviensis*	2.00E-179	94.79%	NR_029287.1
ASV42131	ced92f47e90cfe4f20141ecccbe3d0bd	*Nitrososphaera viennensis*	3.00E-158	93.78%	NR_134097.1
ASV47072	e7208cd1eab8e0bdd5eabbb5beb554fa	*Bacillus clarus*	0	100.00%	NR_115526.1
ASV10264	33520d147bffa680f811c4e5d7a21a1f	*Priestia aryabhattai*	0	100.00%	NR_118442.1
ASV51676	fd95a692130d1f70fbfcb622f1295902	*Alkaliphilus metalliredigens QYMF*	7.00E-80	80.95%	NR_074633.1
ASV40734	c7ca0091732cdf76b704f5df3c86f0ad	*Sphingomonas sediminicola*	0	99.74%	NR_132332.1
ASV1636	086f2152fdb38513ada7b4f3dc53d3eb	*Devosia oryziradicis*	0	100.00%	NR_181423.1
ASV48785	ef7bba86c6cd44346b4b78d369274e86	*Pseudoxanthomonas sacheonensis*	0	99.52%	NR_044293.1
ASV24360	77dd56bf2aedeadc155e7cce44978662	*Nitrososphaera viennensis*	1.00E-171	95.95%	NR_134097.1

Table S14. Tentative identification of fungal ASVs with differential relative abundance (mean >0.5%) in the rhizosphere of winter wheat grown in MP vs. CT soils (FDR < 0.05; Table 7) using SH identifier from UNITE database (version 8.0) with corresponding Accession-number, taxonomy and similarity based on the output file of taxonomic assignment with GALAXY Bioinformatics Platform. These responder ASVs were used for correlation analysis (Fig. 4).

SH No.	Accession No.	Phylum	Class	Order	Family	Genus	Species	Similarity
SH1610194.08FU	GU721832	p__Ascomycota	c__Sordariomycetes	o__Hypocreales	f__Nectriaceae	g__Fusarium	s__Fusarium_sp	100
SH1615796.08FU	MF782744	p__Ascomycota	c__Sordariomycetes	o__Sordariales	f__Chaetomiaceae	g__Chaetomium	s__Chaetomium_sp	99.56
SH2305819.08FU	FJ860810	p__Ascomycota	c__Sordariomycetes	o__Hypocreales	f__Hypocreaceae	g__Trichoderma	s__Trichoderma_piluliferum	100
SH1608147.08FU	KC008744	p__Mortierellomycota	c__Mortierellomycetes	o__Mortierellales	f__Mortierellaceae	g__Mortierella	s__Mortierella_fimbricystis	98.72
SH1513361.08FU	AB540575	p__Ascomycota	c__Sordariomycetes	o__Hypocreales	f__Hypocreales_fam_Incertae_sedis	g__Acremonium	s__Acremonium_persicinum	100
SH2303584.08FU	DQ323441	p__Ascomycota	c__Sordariomycetes	o__Hypocreales	f__Hypocreaceae	g__Trichoderma	s__Trichoderma_petersenii	100
SH2190001.08FU	AY371635	p__Ascomycota	c__Eurotiomycetes	o__Eurotiales	f__Aspergillaceae	g__Penicillium	s__Penicillium_aethiopicum	100
SH1635388.08FU	DQ133892	p__Ascomycota	c__Dothideomycetes	o__Pleosporales	f__Leptosphaeriaceae	g__unidentified	s__Leptosphaeriaceae_sp	100
SH1519091.08FU	AY997067	p__Olpidiomycota	c__Olpidiomycetes	o__Olpidiales	f__Olpidiaceae	g__Olpidium	s__Olpidium_brassicae	99.35
SH2267912.08FU	DQ117444	p__Ascomycota	c__Leotiomycetes	o__Thelebolales	f__Pseudeurotiaceae	g__Pseudogymnoascus	s__Pseudogymnoascus_pannorum	99.55
SH1650287.08FU	HQ630347	p__Mortierellomycota	c__Mortierellomycetes	o__Mortierellales	f__Mortierellaceae	g__Mortierella	s__Mortierella_antarctica	100
SH1648789.08FU	HG936236	p__Ascomycota	c__Leotiomycetes	o__Helotiales	f__Helotiaceae	g__Articulospora	s__Articulospora_sp	100
SH1555467.08FU	MH107898	p__Ascomycota	c__unidentified	o__unidentified	f__unidentified	g__unidentified	s__Ascomycota_sp	97.98
SH1650607.08FU	AF444417	p__Basidiomycota	c__Tremellomycetes	o__Cystofilobasidiales	f__Mrakiaceae	g__Tausonia	s__Tausonia_pullulans	100
SH1510152.08FU	JN206324	p__Mucoromycota	c__Mucoromycetes	o__Mucorales	f__Rhizopodaceae	g__Rhizopus	s__Rhizopus_arrhizus	100
SH1557965.08FU	EF568081	p__Ascomycota	c__Sordariomycetes	o__Hypocreales	f__Stachybotryaceae	g__Stachybotrys	s__Stachybotrys_chartarum	100
SH1552992.08FU	HQ914933	p__Ascomycota	c__Eurotiomycetes	o__Onygenales	f__Onygenales_fam_Incertae_sedis	g__Chrysosporium	s__Chrysosporium_lobatum	100
SH1639424.08FU	EU144472	p__Basidiomycota	c__Agaricomycetes	o__Agaricales	f__Strophariaceae	g__unidentified	s__Strophariaceae_sp	99.6
SH1575137.08FU	AB093521	p__Basidiomycota	c__Microbotryomycetes	o__Sporidiobolales	f__Sporidiobolaceae	g__unidentified	s__Sporidiobolaceae_sp	99.268

Table S15. Characteristics of root-associated soil from winter wheat (cv. Lemmy; EC 59) grown under different long-term agricultural practices (MP- mouldboard plough, CT- cultivator tillage, Ext- extensive fertilization without fungicides and growth regulators, Int- intensive fertilization with pesticides and growth regulators). The soil was sampled at a depth between 0 and 30 cm. Micro- and macro-nutrients were analyzed according to the certified protocols of VDLUFA by AGROLAB GmbH (Leinefelde-Worbis, Germany). Abbreviations: total carbon (TC), total nitrogen (TN), dry matter (DM), organic matter (OM). Values represent means ± standard deviation of four replicates. Means not sharing any letter are significantly different by the Tukey's test ($p \leq 0.05$).

	**MP-Int**			**MP-Ext**			**CT-Int**			**CT-Ext**		
pH	7.3	±0.2	ns	7.5	±0.1	ns	7.1	±0.4	ns	7.2	±0.3	ns
C/N	11.0	±0	ns	10.3	±2.4	ns	10.5	±1.3	ns	11.0	±1.4	ns
**[%]**												
TC	1.82	±0.07	c	1.82	±0.02	c	2.42	±0.08	a	2.09	±0.04	b
TN	0.15	±0	b	0.17	±0.04	ab	0.23	±0.03	a	0.19	±0.02	ab
DM	80.3	±0.96	ns	80.4	±0.32	ns	78.3	±1.36	ns	78.7	±0.99	ns
OM	2.98	±0.08	c	2.95	±0.04	c	4.06	±0.21	a	3.46	±0.08	b
**[mg 100 g^{-1} soil]**												
NO_3-N	0.58	±0.16	a	0.24	±0.04	b	0.50	±0.11	a	0.24	±0.04	b
NH_4-N[#]	0.12	±0.02	ab	0.06	±0.01	c	0.19	±0.07	a	0.10	±0.01	bc
K	16.1	±1.2	b	21.0	±2.0	b	35.6	±4.4	a	31.5	±3.3	a
Mg	10.1	±2.1	ab	8.4	±0.8	b	13.1	±2.6	a	10.5	±2.8	ab
P	15.2	±3.6	b	21.4	±3.1	a	19.3	±4.7	ab	18.4	±4.7	ab
Ca	250	±22	ab	254	±10	a	226	±26	b	237	±21	ab
Fe	4.9	±1.0	ns	4.5	±0.6	ns	5.8	±1.9	ns	4.9	±1.9	ns
**[mg kg^{-1} soil]**												
Cu	2.8	±0.2	ns	2.8	±0.2	ns	2.6	±0.5	ns	2.6	±0.5	ns
Mn	202	±87	ns	171	±69	ns	220	±101	ns	195	±96	ns
Na	12.0	±1.1	ns	12.9	±1.9	ns	12.6	±1.3	ns	12.4	±1.4	ns
Zn	3.9	±0.4	ns	4.2	±0.4	ns	4.1	±0.5	ns	3.7	±0.4	ns

ns: not significant

#: statistical analysis after Tukey's ladder of powers transformation
